# Can You Hear Me Now? Helping Faculty Improve Feedback Exchange for Internal Medicine Subspecialty Fellows

**DOI:** 10.15766/mep_2374-8265.11099

**Published:** 2021-02-17

**Authors:** Sonia Ananthakrishnan, Mara Eyllon, Craig Noronha

**Affiliations:** 1 Assistant Professor of Medicine, Department of Medicine and Director of Student Education, Boston University School of Medicine, Boston Medical Center; 2 Postdoctoral Researcher, Department of Medicine, Boston University School of Medicine, Boston Medical Center; 3 Assistant Professor of Medicine, Department of Medicine and Associate Program Director, Boston University School of Medicine, Boston Medical Center

**Keywords:** Feedback, Faculty Development, Fellow Trainees, Case-Based Learning

## Abstract

**Introduction:**

Feedback is an important tool that describes an individual's performance in a specific activity. Trainees at all levels grow from feedback exchanges to develop knowledge, skills, and attitudes in their specialty. However, there is a dearth of faculty development on providing advanced trainees feedback effectively.

**Methods:**

We designed and delivered internal medicine subspecialty-focused 60- or 90-minute interactive workshop to train faculty to improve feedback exchange with fellows. The workshop included addressing barriers to feedback specific to fellowship, tool and skills for feedback exchange, and case-based skills practice specific to scenarios seen in each subspecialty fellowship program. We utilized surveys of faculty assessing comfort with feedback exchange with fellows before and after the workshop.

**Results:**

We delivered the workshop to two separate specialty sections, gastroenterology and endocrine. Overall, faculty (*N* = 14) self-reported comfort improved significantly from pretest to posttest (*p* < .01). Ten participants’ comfort ratings increased, while four remained the same at posttest. The evaluation identified several common themes as important learning points including labeling feedback, setting expectations around feedback exchange, and identifying elements of high-quality feedback exchange.

**Discussion:**

This workshop for faculty was designed to improve the skills, knowledge, and attitudes related to feedback exchange specifically within an internal medicine subspecialty fellowship training program. Analysis of pre- and postsurvey data demonstrated increased faculty comfort providing fellows-in-training with feedback.

## Educational Objectives

By the end of this activity, learners will be able to:

1. Define the key elements and root components of effective feedback exchange.
2. Describe how to give feedback in specific fellowship scenarios.
3. Increase comfort level with giving feedback to trainees.

## Introduction

Feedback is a valuable tool used to describe an individual's performance in a specific activity, with the intention of guiding future performance.^1–3^ The ACGME defines feedback as ongoing information provided to trainees regarding aspects of performance, knowledge, and understanding.^4^ The ACGME requires faculty to directly observe and evaluate trainees, and frequently provide feedback on trainee performance during each rotation. Within each training level, specialty, and position, there are specific needs and barriers in relation to giving and receiving feedback. Subspecialty fellowship training has unique goals and barriers to feedback exchange that are not seen in other training environments. For example, due to the small sizes of fellowship training programs, there is a higher likelihood that a fellow will have multiple periods of supervision from the same faculty member, compared to residents or medical students. This longitudinal relationship can provide a strong foundation to enhance feedback exchanges. Creating a universal language that includes shared expectations and a culture of feedback exchange is vital in the development and improvement of clinical and professional skills.^5–8^ The ACGME provides guidance to fellows, highlighting five essential features of feedback: timeliness, specificity, balance, recipient reflection, and action plans.^9^

Within the academic realm, there is a large amount of research and literature defining and highlighting the need for feedback exchange among students, resident trainees, and faculty.^5,10–16^ However, there is a dearth of internal medicine fellowship-specific feedback training for faculty. Fellowship is a unique level of training where trainees enter with several years of training, but have increased expectations placed on them as they prepare for the high-stakes stage of independent specialty practice.

Within our institution, ACGME annual survey data from 2015–2018 indicated that anywhere from 25%–80% of internal medicine specialty fellows were not satisfied with the feedback they received. A consistent area where BMC fellowship programs were rated low was in the domain of trainee “satisfaction with feedback after assignments.” Based upon our discussions with fellows and faculty, we identified several barriers to high-quality feedback. Many of the barriers are universal issues identified across the spectrum of medical training.^10,14,17^ Some of the barriers to feedback exchange include lack of time for providing feedback, inadequate physical space for potentially sensitive conversations, competing responsibilities, inexperience with feedback tools, faculty reluctance to give negative feedback, faculty-fellow relationships, and trainee emotional responses to feedback.^5–8,12,14,18–24^

Departmental leadership also noted these deficiencies and supported the development of standardized faculty development workshops to improve feedback exchanges by highlighting principles from the ACGME Milestones Guidebook for Residents and Fellows.^9^

There have been several curricula developed to improve feedback exchanges. For example Moroz et al. demonstrated that faculty development sessions could help create a shared mental model of performance metrics to help guide feedback exchanges.^19^ Schlair et al. published another faculty development model using peer-led observation of feedback encounters.^25^ Furthermore, Sargeant et al. provided evidence that tools such as the R2C2 model could be used to not only deliver feedback but also to explore the reactions to the feedback and understanding what the feedback entailed.^26–28^ Based upon a review of the available literature, there were no curricula focused on internal medicine fellowship training.

To address perceived deficiencies in fellowship-specific feedback exchange, over the last 2 years we have implemented a departmental-wide initiative to improve feedback exchange at all levels including faculty, administration, and trainees, with a specific focus on fellows-in-training. As part of our initiative, we have designed and delivered a subspecialty-focused 60- or 90-minute PowerPoint-based workshop to train faculty to improve feedback exchange with fellows. The sessions focused on tools related to giving feedback and how to handle difficult or sensitive feedback, with the final objective being to increase comfort with giving meaningful feedback. To complement this faculty-directed workshop, we also led feedback workshops for fellows that applied many of the above concepts to these advanced trainees. The fellow-in-training workshops had a larger focus on the importance of feedback in training and developing skills to respond to negative feedback.

## Methods

### Implementation

We conducted this workshop several times with faculty in two department of medicine subspecialty sections, gastroenterology (GI) and endocrine, as both a 60-minute and a 90-minute session. In order to increase the quality of the discussion we felt that it was vital to have sessions for faculty without trainees present. This allowed for more frank and detailed discussions of the goals and barriers to feedback exchange with fellows. Departmental leadership assisted in scheduling workshops as part of normal subspecialty department conference series. Workshops typically included between 10–30 participants depending on the size of the section.

Each workshop was conducted using a facilitator's guide (Appendix A) and general PowerPoint (Appendix B). After participants completed the preworkshop survey (Appendix C), the workshop began with a large-group interactive presentation around barriers to feedback, including those specific to fellowship. The workshop proceeded to review the ask-tell-ask tool for feedback exchange and conclude with group discussions.^29^ The ask-tell-ask tool is validated for providing feedback exchange. We modified the tool slightly by adding an additional step, called add. The add portion allowed for the feedback provider to refine or add to the improvement plan. It was also an opportunity to schedule follow-up feedback exchanges.[Table t1]

Following the large-group discussion, participants were broken into small-groups of four to five participants to discuss four cases (Appendix B). Approximately 3–5 minutes were allotted to small-group discussion followed by 1–2 minutes of large-group debrief per case. The cases included specific scenarios seen in each subspecialty fellowship program allowing for small-group role-plays, or skills practice, for the faculty to practice giving feedback in challenging situations with trainees. For example, we reviewed cases where fellows and faculty interfaced in the endoscopy suite for the GI section faculty. All cases can be modified according to the subspecialty. Faculty, particularly medical education leads (e.g., fellowship program directors), from each subspecialty were contacted prior to each session in order to ensure the cases represented common feedback scenarios specific to each subspecialty. At the end of the workshop we asked all the participants to complete a postworkshop survey (Appendix D).

In order to implement this workshop locally, the following materials and local resources were necessary:

• Workshop leader(s).
• Support staff to schedule workshops.
• AV equipment to project slides.
• Sufficient space to accommodate both large- and small-group discussions.
• Buy-in and support from the leadership in department of medicine and subspecialty sections.

### Evaluation Method

We evaluated the workshop by measuring participant comfort with delivering feedback on a 5-point Likert scale (1 = *extremely uncomfortable*, 5 = *extremely comfortable*) both before and after the workshop, and asked what they took away from each session. We initially administered a seven-question retrospective pre/postsurvey for the GI section and asked two open-ended questions regarding learning points from the session. After leading the workshop for GI faculty, we refined the survey instrument and created a separate preworkshop survey (Appendix C) and postworkshop survey (Appendix D) which were administered to the endocrine faculty who participated in the subsequent workshop.

Survey data were analyzed using the nonparametric sign test to compare faculty comfort level providing feedback to fellows before the workshop to after participating in the workshop. The sign test is appropriate for analyzing Likert-type data and small sample sizes. The two open-ended questions were analyzed using thematic analysis. The data from each section were combined as the sample size for each specialty was small.

## Results

After presenting to approximately 18 endocrine and GI section faculty, 14 participants completed the pre- and postworkshop survey. Overall, faculty's self-reported comfort improved significantly from pretest (*M* = 3.3, *SD* = .8) to posttest (*M* = 4.1, *SD* = .4; *p* < .01). The results of the sign test demonstrated that 10 participants’ comfort ratings increased, while four remained the same at posttest. We did not observe any negative differences indicating that no participants comfort decreased after participating in the session.

After reviewing the open-ended question responses, we identified four common themes emphasizing what the learners gained from the session: expectations, labeling feedback as feedback, timing, and providing specific and constructive feedback (Table). These themes demonstrated key skills that learners gained during the curriculum. These skills facilitated the provision of feedback to fellows that was both concrete and timely to help improve performance.

**Table. t1:**
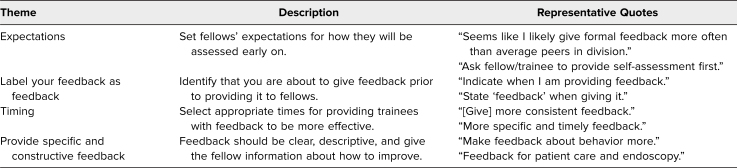
Common Themes From Postworkshop Survey

## Discussion

This faculty-focused workshop utilized a combination of didactic teaching, large- and small-group discussions, and case-based skills practice to improve the skills, knowledge, and attitudes related to feedback exchange specifically within an internal medicine subspecialty fellowship training program. Based upon analysis of pre- and postworkshop survey data, participants in our workshops have demonstrated increased comfort providing fellows-in-training with feedback. A large number of participants highlighted the ask-tell-ask-add model and key elements of feedback as key takeaway points.

One strength of this 60- or 90-minute workshop was that it can be easily tailored to specific specialties with slight modifications to the case discussions. The workshop included a discussion of the core elements of feedback exchange, through a discussion related to the potential barriers to feedback exchange. Having a workshop cases dedicated to individual specialties allowed for rich and open discussions on topics uniquely specific to the participants. It also allowed for sections to create internal improvement plans for feedback exchange.

The majority of the cases were crafted to highlight real life, common themes in feedback exchange in each specialty. We also included cases that featured more sensitive issues and often more challenging feedback exchanges. For example, we had cases related to body odor or dress code discussions. This approach enabled faculty with different levels of expertise in medical education to engage in the session, avoiding overwhelming novice medical educators while also fine tuning skills for the advanced educators.

The main goals of the workshop were to improve attitudes towards feedback exchange, increase knowledge and utilization of a standardized feedback tool, and practice feedback skills. Overall, faculty self-reported comfort with given feedback to fellows improved significantly from preworkshop to postworkshop.

Our workshop is one form of curricula that can be used for faculty skills development focused specifically on feedback exchange within internal medicine fellowship training. This curriculum can be utilized by internal medicine fellowship programs to improve feedback exchange within their institutions by providing guided discussions around feedback and utilizing specific tools to facilitate feedback exchanges.

Our workshop provided opportunities to discuss several elements related to feedback including barriers, goals, and an introduction of a tool to provide feedback. The workshop was intended to provide training for a wide spectrum of faculty. Due to time limitations, the cases utilized focused on the most common scenarios seen during feedback exchange in each specialty. There was limited time to discuss uncommon but potentially more difficult scenarios. These workshops were intended to be presented as a group and may not be as effective for independent study. We do believe that the format of these workshops could be modified to easily fit into a virtual learning environment.

Our data are currently limited to only surveys provided during the workshop, so it was unclear if skills training and attitudes developed during the workshop will persist over time. The overall results were underpowered by the small sample sizes of each group. Given the relatively small number of faculty in each section, it requires further study to determine if there is variance in terms of impact based upon factors such as specialty, experience, or gender. For the GI workshop only a retrospective pre/postworkshop survey was administered and participants were asked to recall their comfort delivering feedback prior to participating. Finally, our evaluation survey was somewhat limited given the time constraints we had to conduct and evaluate the session. Future iterations of this project should include additional evaluation items, including specific questions about intention to change behavior and changes in knowledge about giving feedback.

We hypothesize that presenting the workshop may have some effect on the culture and attitudes related to feedback exchange within a section, but we have not studied this effect. We are considering ways to examine the long-term effect of our curriculum on culture and behaviors. Ideally we would use direct observation in both pre- and postworkshop time periods to examine the impacts of the workshops, but this would be impractical given our limited resources and multiple variables related to feedback exchange such as frequency, location, and delivery methods. Furthermore, we have presented this workshop to a subset of the sections in our department of medicine, so it is unclear if it would have the same results for participants from all specialties. Finally, this workshop has only been provided at a single medical center and it is unclear if the same curriculum would have similar benefit at other institutions. Ultimately, positively impacting the culture of feedback will require educators to be focused on improving knowledge, skills, and attitudes around the feedback exchange, but also learners feeling more empowered to seek and implement the feedback they receive.^16^

There are several potential future opportunities to transform the workshop based on the needs of each section or department. The cases could be modified to include topics such as gender- or race-related microaggressions, research-specific issues, or include cases submitted by participants. Case-based skills practice or role-playing may also be a valuable component of future iterations of this workshop. Future directions may include follow-up workshops presented several months after the initial workshop to discuss new cases and review participants’ experiences with feedback exchange.

## Appendices

Facilitators Guide.docxPowerPoint.pptxPreworkshop Survey.docPostworkshop Survey.doc
All appendices are peer reviewed as integral parts of the Original Publication.
