# Supplementary material for: Can You Hear Me Now? Helping Faculty Improve Feedback Exchange for Internal Medicine Subspecialty Fellows
Source: MedEdPORTAL. 2021 Feb 17;17:11099. doi: 10.15766/mep_2374-8265.11099 (PMC7901254; doi:10.15766/mep_2374-8265.11099)
Supplement: Supplementary file 1 — Facilitators Guide.docxPowerPoint.pptxPreworkshop Survey.docPostworkshop Survey.doc [file mep_2374-8265.11099-s001.zip › A. Facilitators Guide.docx]

**Can You Hear Me Now- Helping Faculty Improve Feedback Exchange for Internal Medicine Sub-specialty Fellows**

**Workshop Facilitators Guide**

**Materials Needed:**

- Computer with Microsoft PowerPoint capabilities, projector, audiovisual capabilities,
- Setting: Private room without interruptions. Chairs arranged to allow for small group discussions facing the front of the room.
- Handouts: pre and post workshop surveys (if desired for feedback on the sessions)
- Pre-session preparation:
  - Adapt sample cases for each subspecialty. For example, if presenting to gastroenterology faculty, you may wish to include cases in the appropriate settings (endoscopy suite) and of appropriate subject matter (consult). We suggest communicating with medical education leads from each sub-specialty section to clarify relevant case scenarios
  - If possible, a brief needs assessment can include ACGME annual survey and/or internal program assessments.

**Recommended leaders/facilitators:**

- Session leader(s): Departmental feedback champions were utilized in our workshop. If these are not available, we advise asking medical education leads in each section/department to lead the workshops.

**Session Outline:**

The session is designed to take 60-90 minutes depending on availability, using the following suggested timeframe (can adapt as needed to timing constraints):

- Introduction, Learning Objectives, Agenda (10 minutes)
- Purpose and Definitions (5 minutes)
- Small Group Activity #1- Goals and Barriers (5 minutes discussion and 5 minutes reporting)
- Key Characteristics/Elements of Feedback Large Group Discussion (5 minutes)
- Ask-Tell-Ask-Add Tool Introduction (5 minutes)
- Labeling Feedback and Content Areas for Feedback (5 minutes)
- Managing Negative Feedback Reactions (5 minutes)
- Small Group Cases with Large Group Debrief (20 minutes/5 minutes per case)
- Concluding Remarks and Questions (5 minutes)

**Objectives:**

By the end of the session, learners will be able

1. Define the key elements and root components of effective feedback exchange
2. Describe how to give feedback in specific fellowship scenarios
3. Increase comfort level with giving feedback to trainees

**Detailed Explanation of Session Components:**

**Audience:**

Workshop sessions are held as part of routine section/department faculty meetings or grand round series. For the purpose of this workshop, we aimed to target faculty who work with fellows in internal medicine sub-specialty fields. Optionally, can also be given as separate faculty development sessions. The optimal size for this workshop is somewhere between 20-30 total participants which allows for multiple small groups for the discussions. The workshop can be also be given for small groups of faculty, but if there are less than total 6 participants presenters may consider converting the small group discussions to one large group discussion for the entire workshop.

**Pre-Workshop:**

As participants enter the session, they can be provided with pre- and post-session survey, if desired. If needed participants should be asked to complete the pre-session survey in the few minutes prior to the start of the workshop.

**Introduction, Learning Objectives, Agenda (10 minutes):**

The workshop starts with an introduction of workshop presenters, a brief review of the learning objectives, and presentation of the session agenda.

**Purpose and Definitions (5 minutes):**

Presenters review importance of feedback and any available data related to satisfaction and/or quality of feedback. This data can be obtained from ACGME surveys or potentially internal program assessments. The presenters can also discuss how feedback exchange is integrated in their specific roles.

Impact of feedback exchange on stakeholders is reviewed. A particular emphasis is made that faculty provide the foundation to drive cultural change around feedback exchanges as the institutional memory. Facilitators should highlight that ultimately, positively impacting culture of feedback will require educators to focus on improving knowledge, skills, and attitudes around feedback exchange but also learners feeling more empowered to seek and implement the feedback they receive.

The presenters go on to discuss the differences between assessments and feedback. We highlight that an ideal performance evaluation should include multiple feedback exchanges and summative assessment(s). With formative feedback at multiple time points along the way, this allows learners to iteratively improve their performance PRIOR to the end of an assignment, thus allowing the summative feedback or assessment to reflect the improvement. The group as a whole is prompted to discuss their perceived definition of feedback.

**Small Group Activity #1- Goals and Barriers (5 minutes discussion and 5 minutes reporting):**

The workshop presenters should introduce the next section which is a small group exercise. We suggest groups of 4-5 people. The presenters should advise the small groups to discuss specific goals and barriers to feedback exchange during internal medicine sub-specialty fellowship. In the presenters’ experience, most of the discussion has focused on barriers to feedback exchange. After the discussion show the slide labeled **Common Challenges to Giving Feedback**. Presenters should highlight that in medicine sub-specialty sections, small sections/programs and the fact that fellows may be future colleagues are 2 distinct challenges specific to the faculty-fellow feedback dynamic.

**Key Characteristics/Elements of Feedback Large Group Discussion** **(5 minutes):**

At this point many of the key elements and characteristics of high quality feedback exchange may have been identified in prior discussions. Session leaders should reframe or reword previously mentioned elements to match the 5 major elements on the slide. Presenters should invite participants to identify other elements not previously noted. For elements previously discussed, presenters can discuss examples and strategies around these elements.

Examples for characteristics/elements of feedback:

**Set Expectations:** Faculty participants should be reminded to set expectations for fellows on day 1 of each rotation. Faculty should elicit personal learning goals from fellows on a regular basis.

**Timely:** This is where presenters should highlight the importance of providing feedback in real time. In addition, “timely” also suggests avoiding spending too much time giving feedback i.e. the pitfalls of feedback overload in a short period of time

**Specific:** Presenters can review strategies about collecting specific observations of behaviors. For example, taking brief notes on observations during clinical encounters or presentations.

**Behavioral Based:** Specific examples of personality based vs behavioral based feedback should be provided. A strong emphasis should be placed on avoiding any personality focused feedback. Feedback should be about the behavior, not about the person. Identifying perceptions of behavior is a tool to describe an observation.

The presenters can choose to model the difference between behavioral based and personality based feedback. For example, instead of wording the feedback “you are too arrogant” it can be restated as saying “from my view, the tone of your voice and your body language may be perceived as arrogant by the residents and students.” Refer to slide with examples of personality based feedback.

**Direct Observation:** It is important to highlight that high quality feedback exchange should be based on directly observed behaviors. That being said, faculty should be reminded that they are in a position that they are in a position to deliver feedback related to behaviors they did not directly observe. For example, if the faculty receives information about a difficult encounter between a nurse and a fellow, the faculty member should be encouraged to discuss that feedback with the fellow.

**Ask-Tell-Ask-Add Tool Introduction (5 minutes):**

*****This is a modification made to original tool from French JC, Colbert CY, Pien LC, Dannefer EF, Taylor CA. Targeted Feedback in the Milestones Era: Utilization of the Ask-Tell-Ask Feedback Model to Promote Reflection and Self-Assessment. J Surg Educ. 2015;72(6):e274-e279. doi:10.1016/j.jsurg.2015.05.016

The authors created an ADD portion to the “ask-tell-tell" tool as a modification to summarize the discussion and co-create a follow-up plan.

The presenters will introduce the Ask-Tell-Ask-Add tool for feedback exchange.

**Ask-** The 1^st^ step in the tool helps frame the discussion and allows the trainee to reflect on and assess their own performance. The planned feedback discussion may vary depending on how accuracy and quality of the trainee’s self-assessment.

**Tell-** The 2^nd^ step involves the feedback provider discussing their impression of the behavior in a timely and specific manner. Faculty should be advised to address a maximum of 1-2 areas for improvement to avoid feedback overload.

**Ask-** The 3^rd^ step focuses on asking the fellow to start developing a specific improvement plan. This step also serves to evaluate the trainee’s understanding of the feedback.

**Add-** The provider can refine or add to the improvement plan. This step is an opportunity to highlight the positives of the observed behavior. Faculty should be advised that follow-up related to the feedback exchange should be scheduled, for example “let’s review your performance in one week.”

**Labelling Feedback and Content Areas for Feedback (5 minutes):**

This portion of the workshop focuses on the importance of labeling feedback and reviewing potential topics to provide feedback on.

Presenters should emphasize that faculty should use some variant of the words “let me give you feedback” as a way to introduce the interaction and to prime all parties for the exchange. Although this has not been quantified, we believe that trainees often do not perceive and recall that they received feedback unless the exact word “feedback” is used when starting the conversation. Feedback can sometimes be confused for teaching.

The second portion of this section starts with an open discussion where the presenters ask the audience to provide examples of topics or specific areas where trainees can be given feedback. After generating a short list, the session leaders will present the list of potential topics for feedback. Depending on specific areas of concern for each section, the presenters may want to want to focus on specific topics such as professionalism or staff interactions that are not usually considered in feedback exchange for clinical training. For example, for trainees entering the workforce, email correspondence and completion of administrative duties, such as filing licensing forms, may be a valuable topic of feedback exchange.

**Addressing Negative Feedback (5 minutes):**

The presenters will review the importance of communicating negative formative feedback and highlight challenges and best practices to manage negative reactions to feedback.

Separate workshops on feedback exchange for trainees that include coaching around receiving and responding to difficult feedback can be provided by either the presenters or within each section. This may enable trainees to avoid defensive responses so as to maintain longitudinal lines of feedback exchange.

The presenters should remind faculty participants that clinical competency and potentially patient lives are on the line, thus highlighting the importance of conveying formative feedback despite inherent difficulties in doing so. Challenges (derived from slide “Common Challenges to Giving Feedback”) that are relevant to the specific audience can be highlighted. For example highlighting challenges in conveying difficult feedback to a fellow who may shortly join the department as junior faculty.

The slides “Keys for Trainees to Respond to Negative Feedback” highlight important skills for trainees to effectively respond to negative feedback. Departments and educational leaders should be encouraged to teach these strategies to trainees. In discussing negative feedback, faculty can provide high quality feedback with specific, behavioral based examples. Faculty should answer questions about the feedback in order to allow the trainees to clarify the issue the feedback pertains to.

The presenters should discuss that training programs can aim to create a learning environment that fosters self-reflection. The presenters should remind participants to be open to varied responses to feedback. Within the feedback exchange, this may include real-time self-reflection immediately following feedback exchange. Additionally or alternatively, this may also include a brief follow-up meeting to review the feedback again, in order to allow the trainee sufficient time to process the feedback. As with all feedback, the exchange of difficult feedback should consistently include an action plan with a future plan to follow-up on the feedback and assess for improvement.

**Small Group Case Discussions (20 minutes):**

In our experience, this is the most valuable portion of the workshop, thus sufficient time should be allotted to allow for small group discussion and then a brief large group debrief of each case. Depending on the size of the audience, the participants can be asked to discuss each case in groups of 4-5. Approximately 3-5 minutes can be allotted to small group discussion followed by 1-2 minutes of large group debrief per case. Participants are asked to specifically consider the following questions in their small groups:

- Have you been in this situation before?
- Would you give feedback normally in this situation?
- How would you approach giving feedback?

The small group discussions often reinforce previously mentioned issues or barriers to feedback exchange such as lack of perception of feedback exchange (i.e. feedback was not labeled) or difficulties with delivering negative feedback in a small section. In the large group debrief, participants are asked to provide specific language they would use in the feedback exchange.

Depending on how robust the discussion is, you may not get through all of the questions for each case. Presenters can modify what cases they use or the length of discussion based on time allotted for the session and the discussion generated by previous teaching points.

Skills Practice Small Group Modification:

If desired, facilitators can have small groups utilize the cases for skills practice with one participant acting as the trainee and another participant being the feedback giver. Other members in the small group can act as observers. If the case discussions are utilized as small group discussions, there should be time reserved for a debrief after each feedback exchange to highlight the positives and negatives of the discussion.

Specialty specific case development:

Ideally, each case is tailored to the audience or specialty. For example, Case 2 can be altered to feature thyroid biopsies if the workshop is being conducted with the Endocrinology section, rather than discussing colonoscopies with the Gastroenterology section. As trainees work in different settings (clinic, procedure suite, research lab), we encourage presenters to utilize cases that span a variety of trainee experiences. Presenters should attempt to reach out to educational leadership of each section in advance of the workshop presentation, in order to identify key scenarios that will be relevant for case-based discussions.

Sensitive Case Discussion- Case 4

The presenters can consider reviewing at least one case that touches on a sensitive issue. These typically touch on behaviors or practices that cause an uncomfortable social environment such as lack of professional attire (example inappropriate shirt). The cases provide a venue for section or departmental level discussions about topics that may be culturally sensitive. These cases also allow the presenters to highlight the importance of partnering with trainees to ensure that the learning environment is safe and supportive (See slide Tips for delivering sensitive feedback). For certain topics, it might be appropriate to have faculty of the same gender deliver sensitive feedback (personal hygiene); other topics are well suited to address a group of trainees (professional attire). Group discussions can also reinforce institutional and section specific standards and policies.

An open discussion can create the opportunity to reinforce guidelines or potentially create policies around these topics.

**Concluding Remarks and Questions (5 minutes):**

**Closing:**

The workshop can be concluded with a brief review of the slide labeled “Key Characteristics and elements of feedback”.

The presenters are encouraged to reinforce the importance of providing feedback to fellows, a frequently overlooked training group. Acknowledgement of fellow- specific barriers and topics for feedback may be included in the closing discussion.

**Post-Workshop:**

If desired, a post-workshop survey can be administered. As participants leave the session, the pre- and post-session surveys can be collected.

**Notes on format and timing:**
The workshops have been presented at section faculty meetings, grand rounds, or as part of faculty development series. The time allocated has been anywhere between 60 to 90 minutes, with the pace of the session being modified according to the discussion generated. The number of cases discussed at the end can be modified as needed.

**Virtual Teaching Modifications:**

This workshop can easily be converted to a virtual model. Pre and post workshop surveys can be delivered electronically. In order to facilitate small group discussions, presenters would need to utilize virtual platforms that allow for breakout rooms or group discussion rooms. The majority of the workshop would not need to be modified for presentation in the virtual world.
