# Supplementary material for: Can You Hear Me Now? Helping Faculty Improve Feedback Exchange for Internal Medicine Subspecialty Fellows
Source: MedEdPORTAL. 2021 Feb 17;17:11099. doi: 10.15766/mep_2374-8265.11099 (PMC7901254; doi:10.15766/mep_2374-8265.11099)
Supplement: Supplementary file 1 — Facilitators Guide.docxPowerPoint.pptxPreworkshop Survey.docPostworkshop Survey.doc [file mep_2374-8265.11099-s001.zip › B. PowerPoint.pptx]

## Slide 1
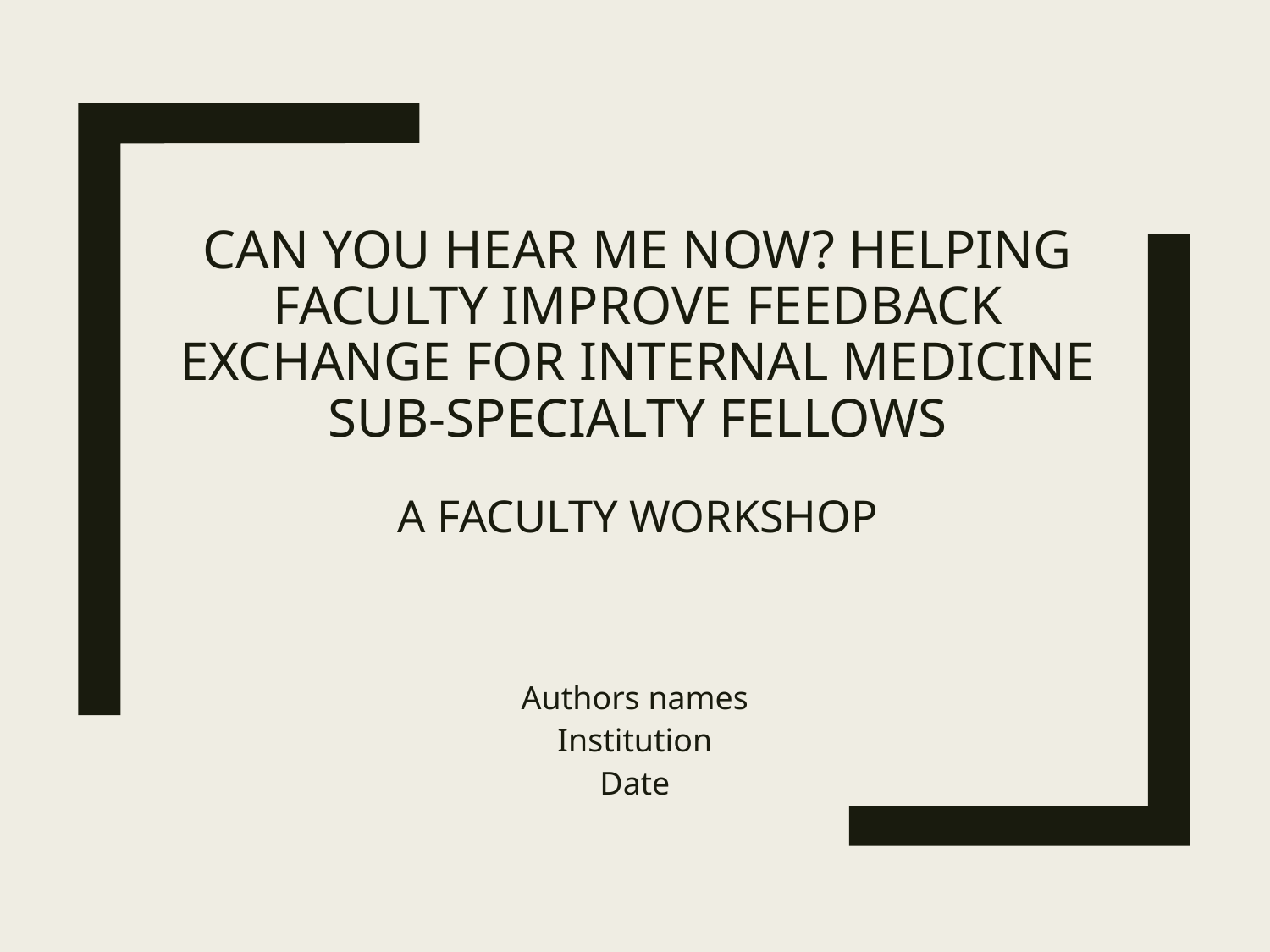

# Can You Hear Me Now? Helping Faculty Improve Feedback Exchange for Internal Medicine Sub-specialty Fellows A Faculty Workshop
Authors names
Institution
Date

## Slide 2
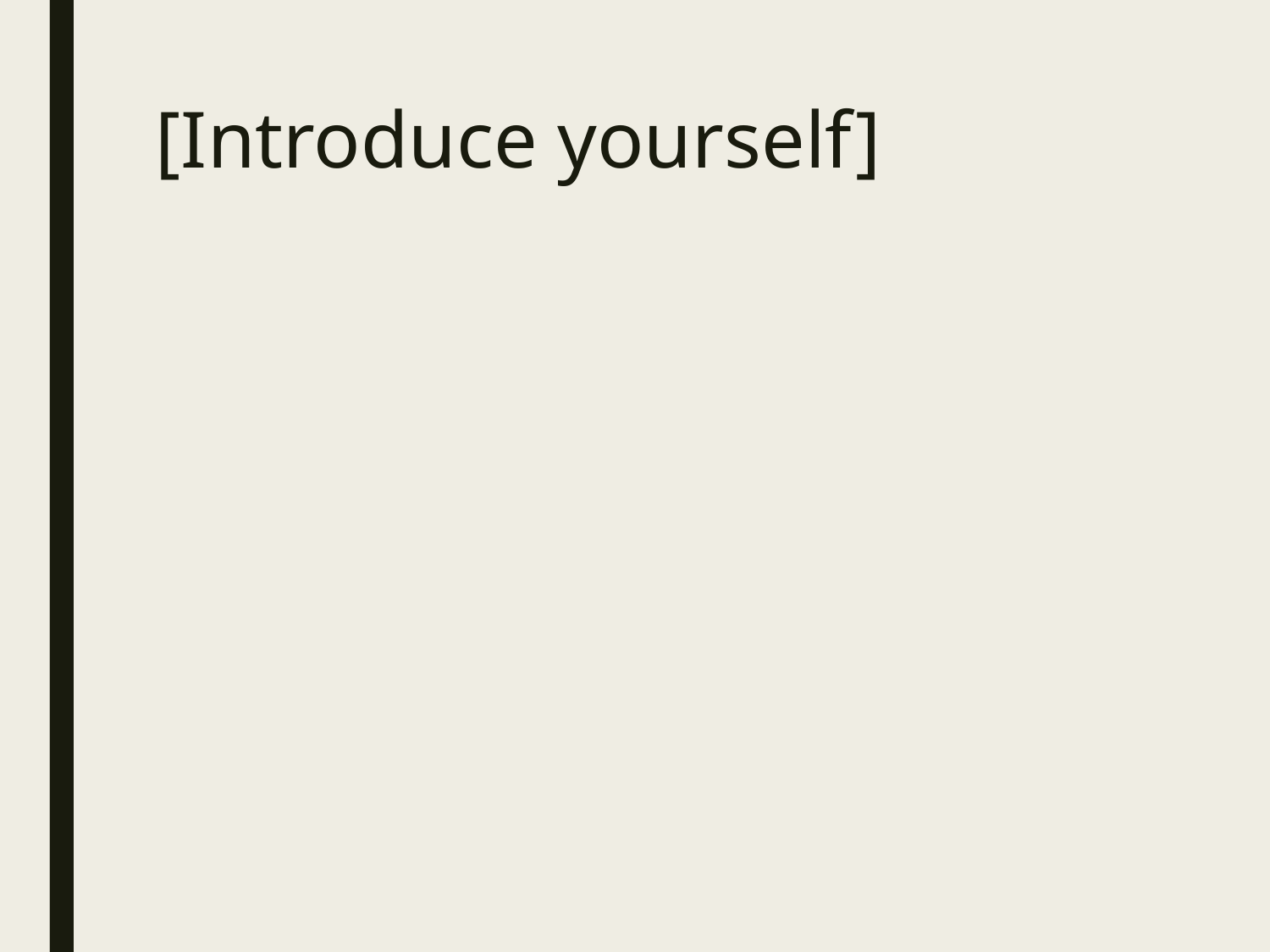

# [Introduce yourself]

## Slide 3
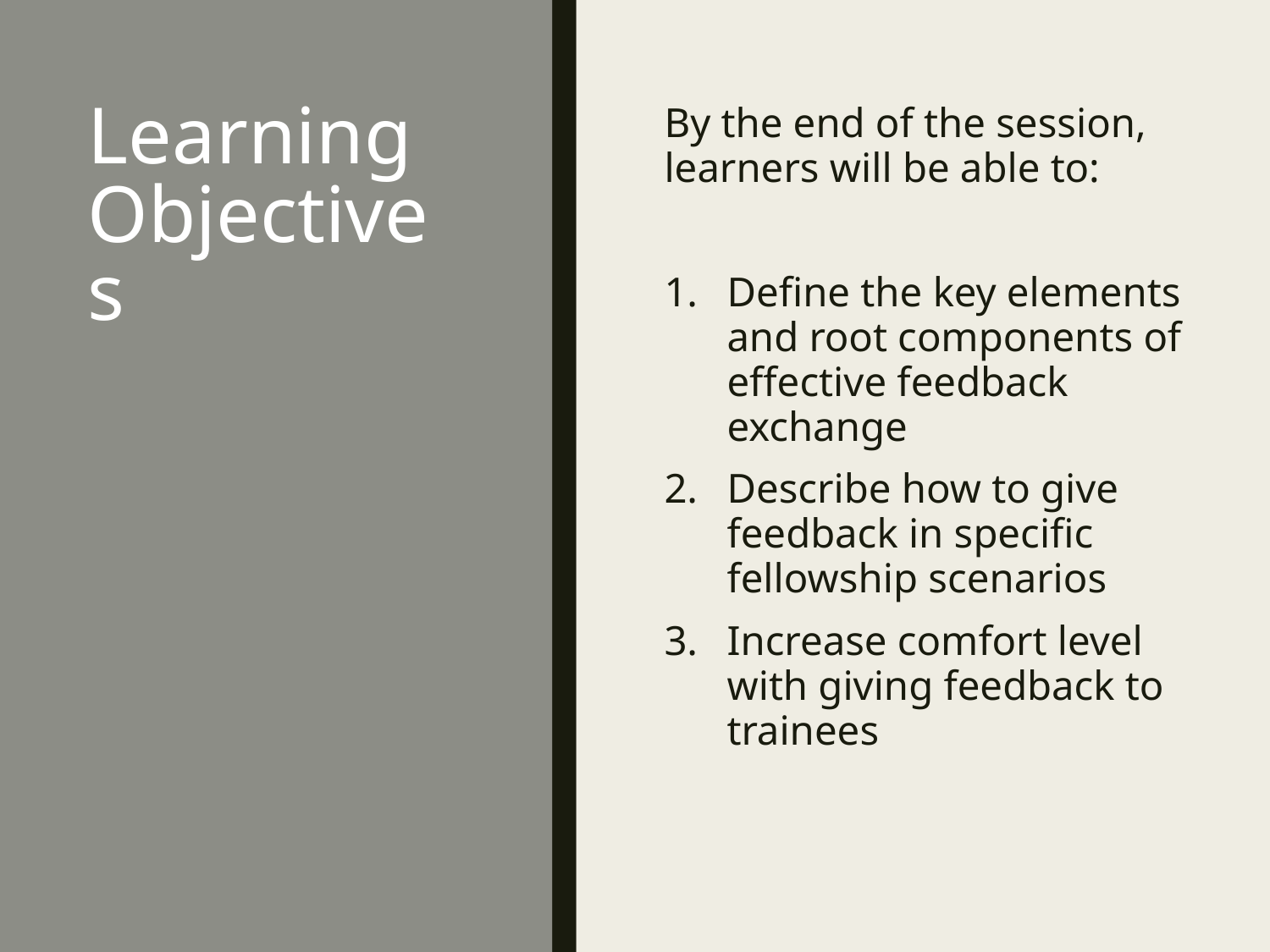

# Learning Objectives
By the end of the session, learners will be able to:
Define the key elements and root components of effective feedback exchange
Describe how to give feedback in specific fellowship scenarios
Increase comfort level with giving feedback to trainees

## Slide 4
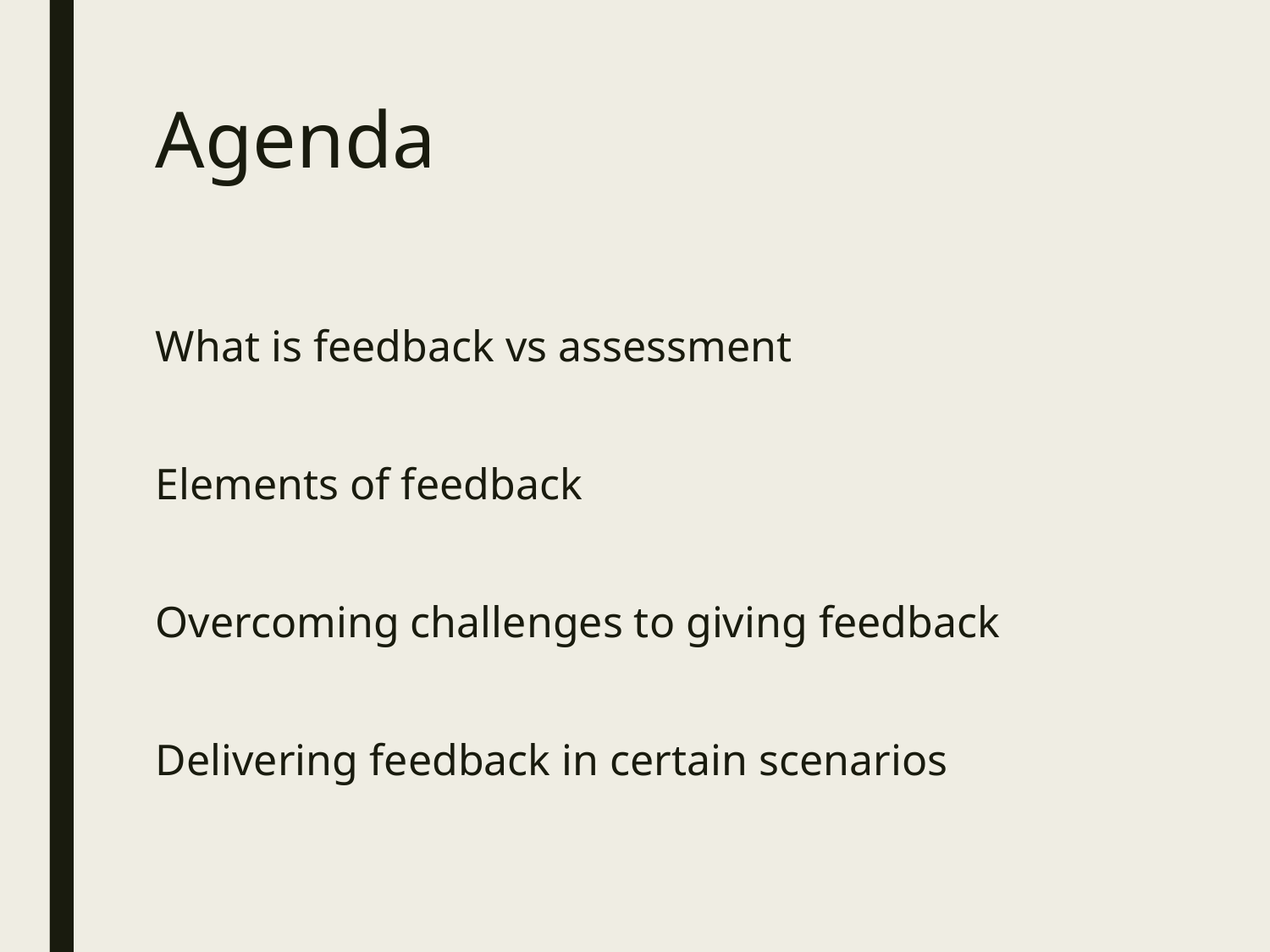

# Agenda
What is feedback vs assessment
Elements of feedback
Overcoming challenges to giving feedback
Delivering feedback in certain scenarios

## Slide 5
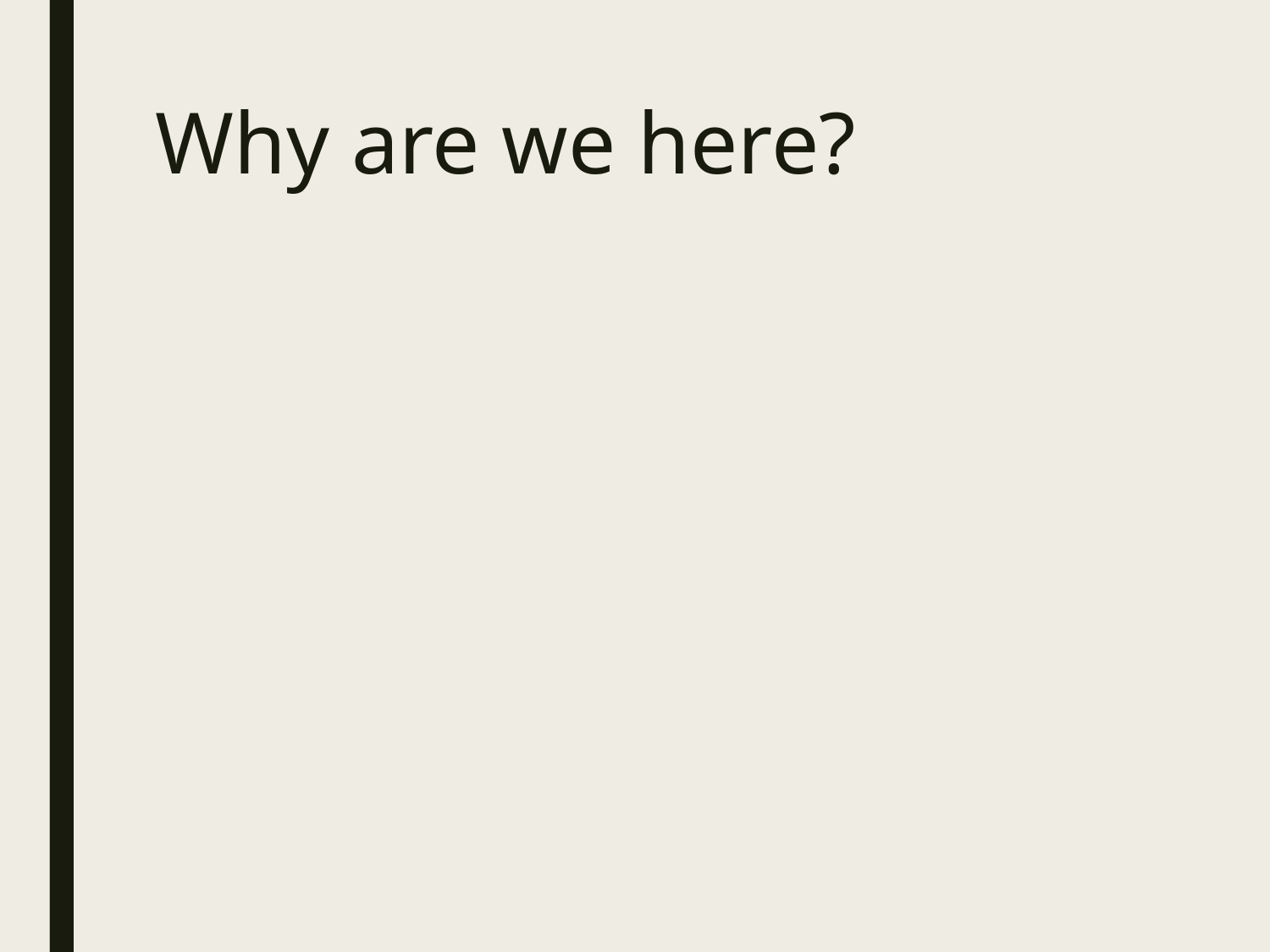

# Why are we here?

## Slide 6
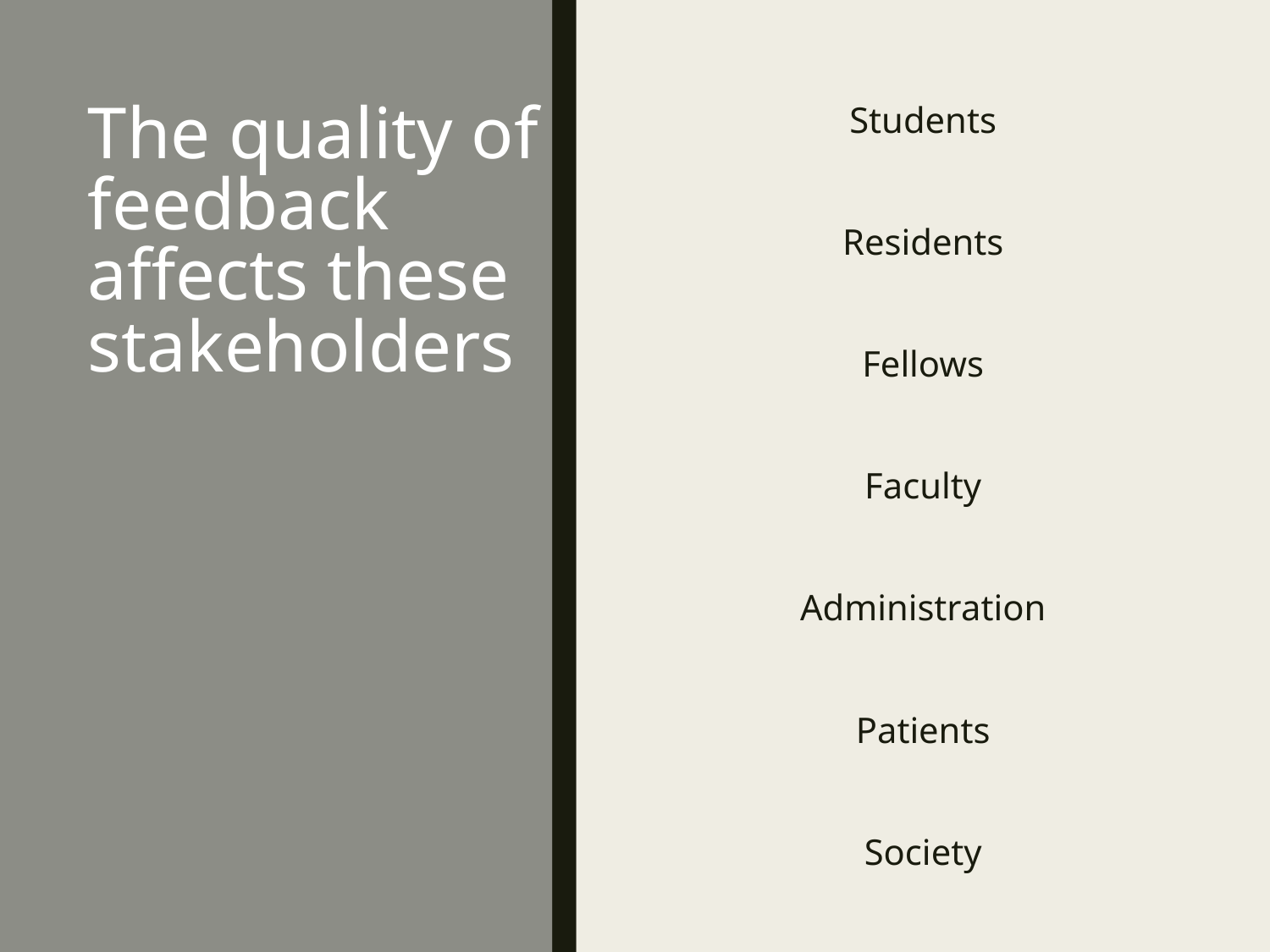

# The quality of feedback affects these stakeholders
Students
Residents
Fellows
Faculty
Administration
Patients
Society

## Slide 7
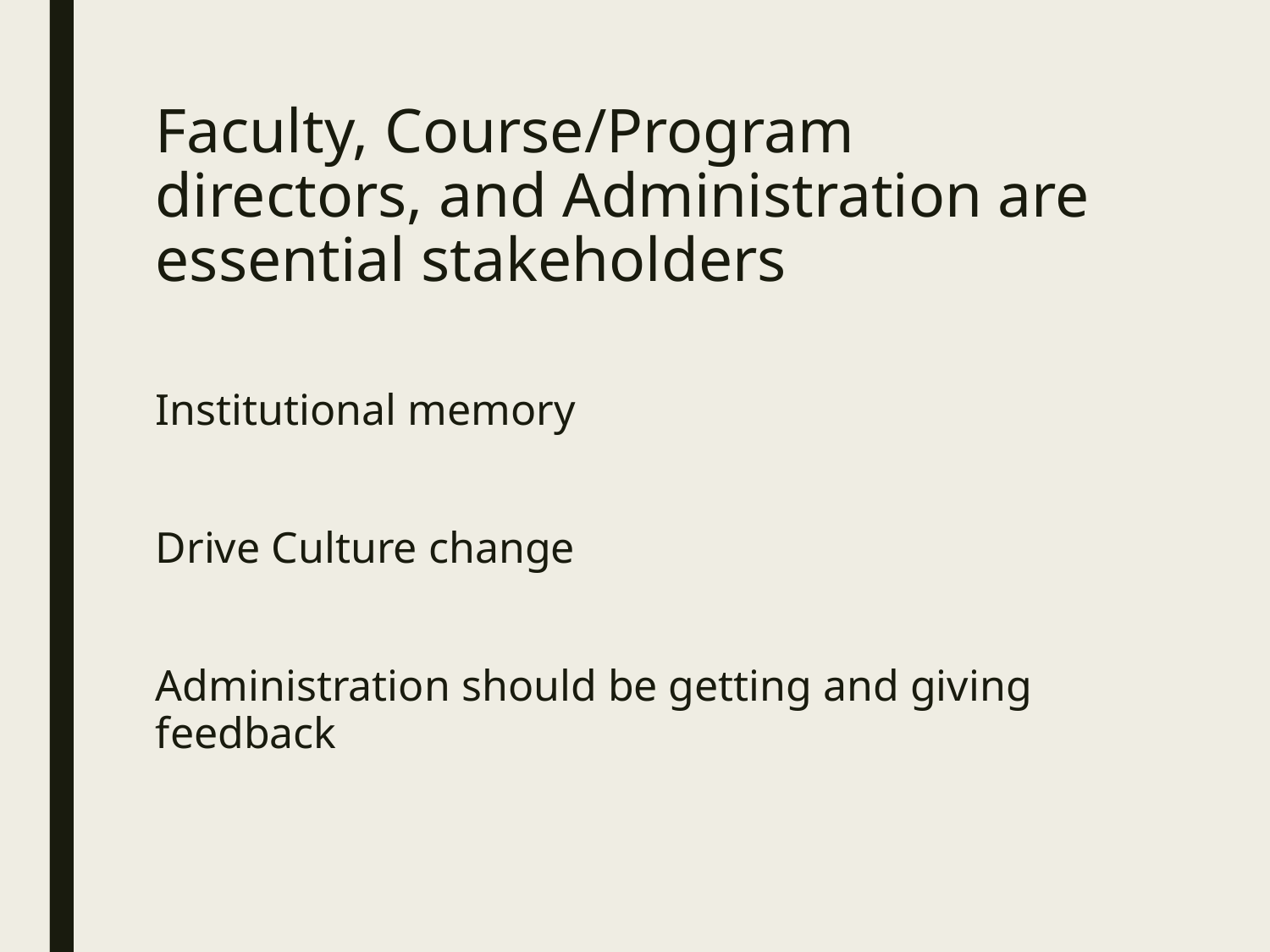

# Faculty, Course/Program directors, and Administration are essential stakeholders
Institutional memory
Drive Culture change
Administration should be getting and giving feedback

## Slide 8
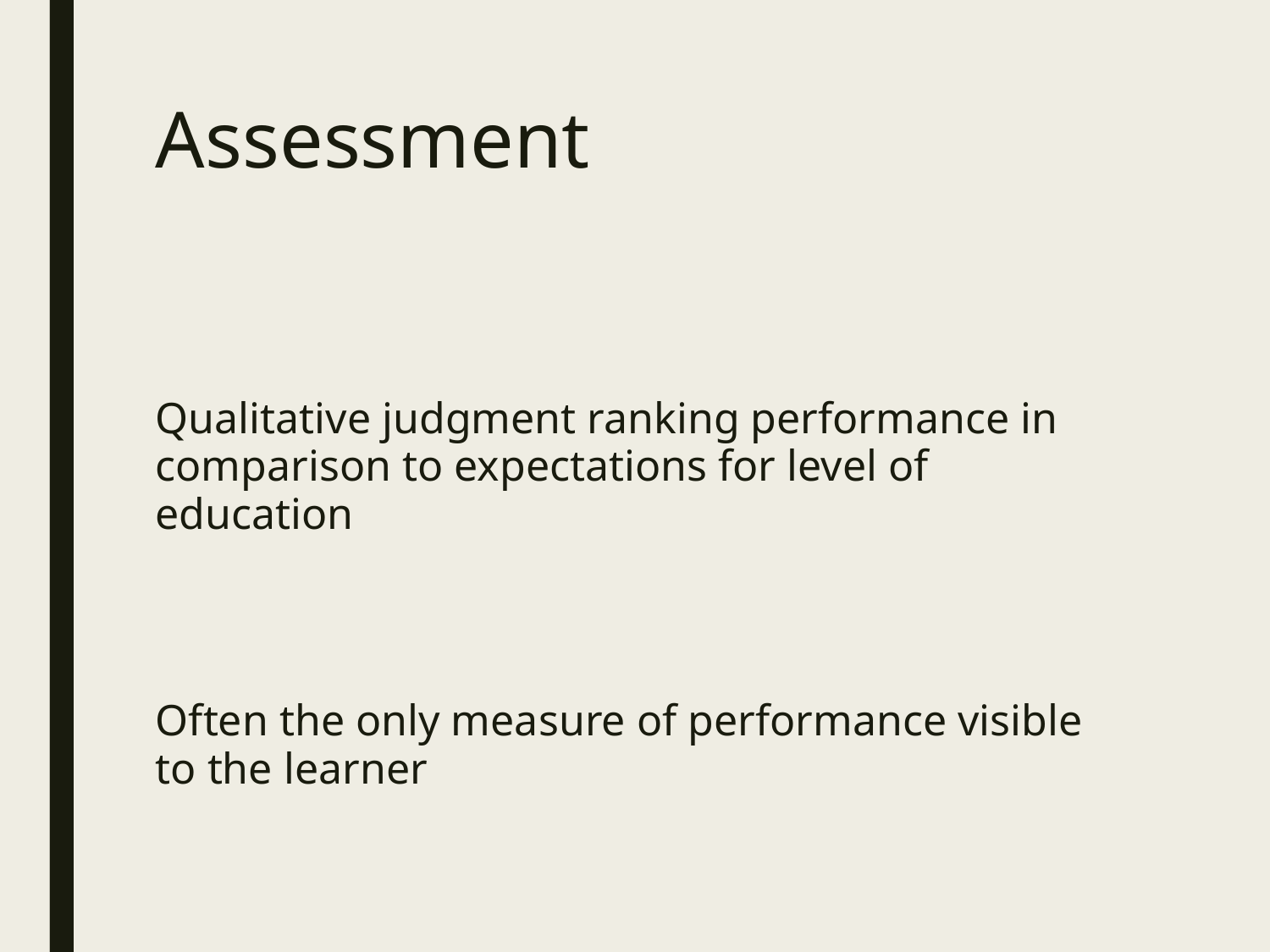

# Assessment
Qualitative judgment ranking performance in comparison to expectations for level of education
Often the only measure of performance visible to the learner

## Slide 9
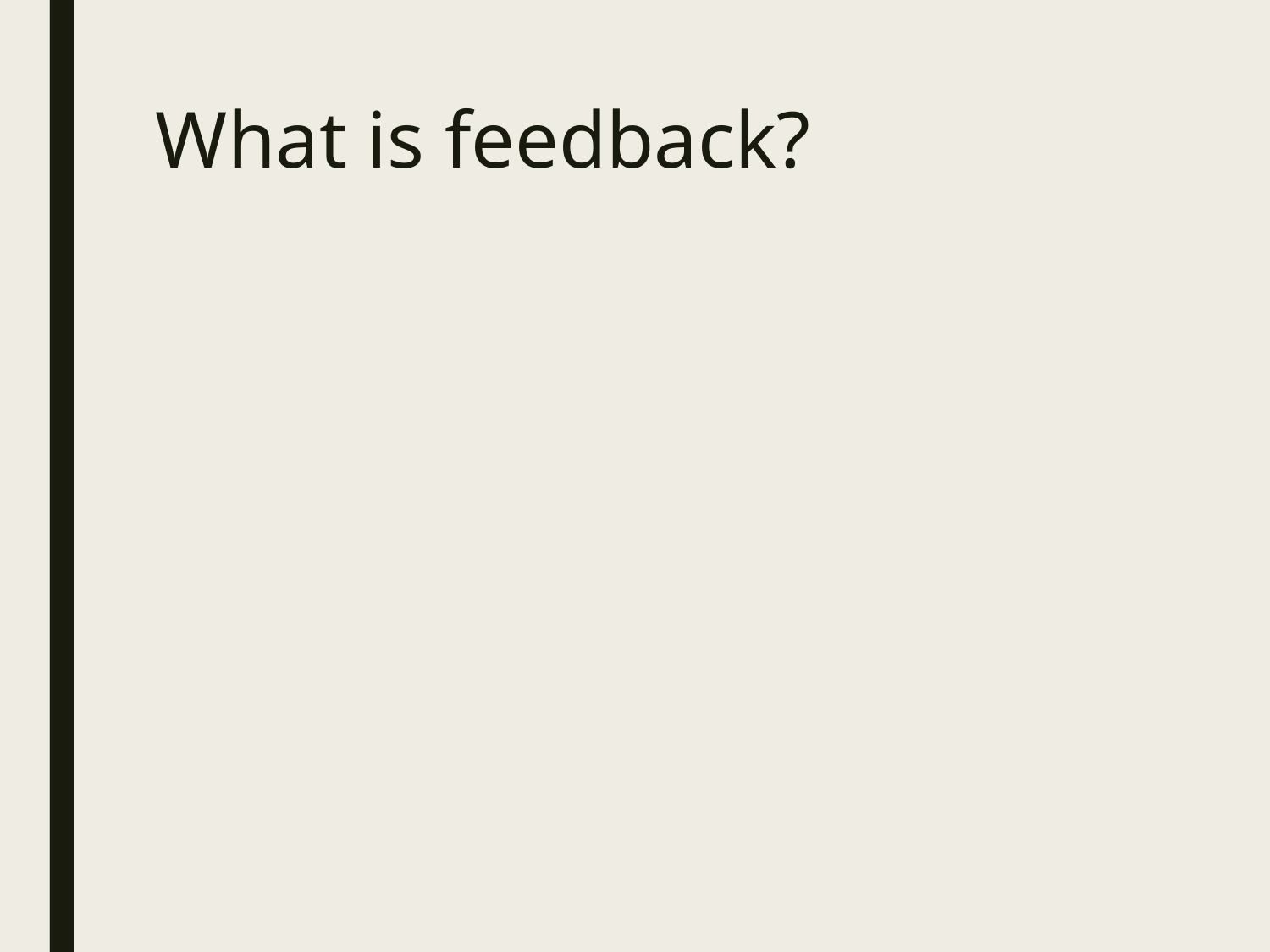

# What is feedback?

## Slide 10
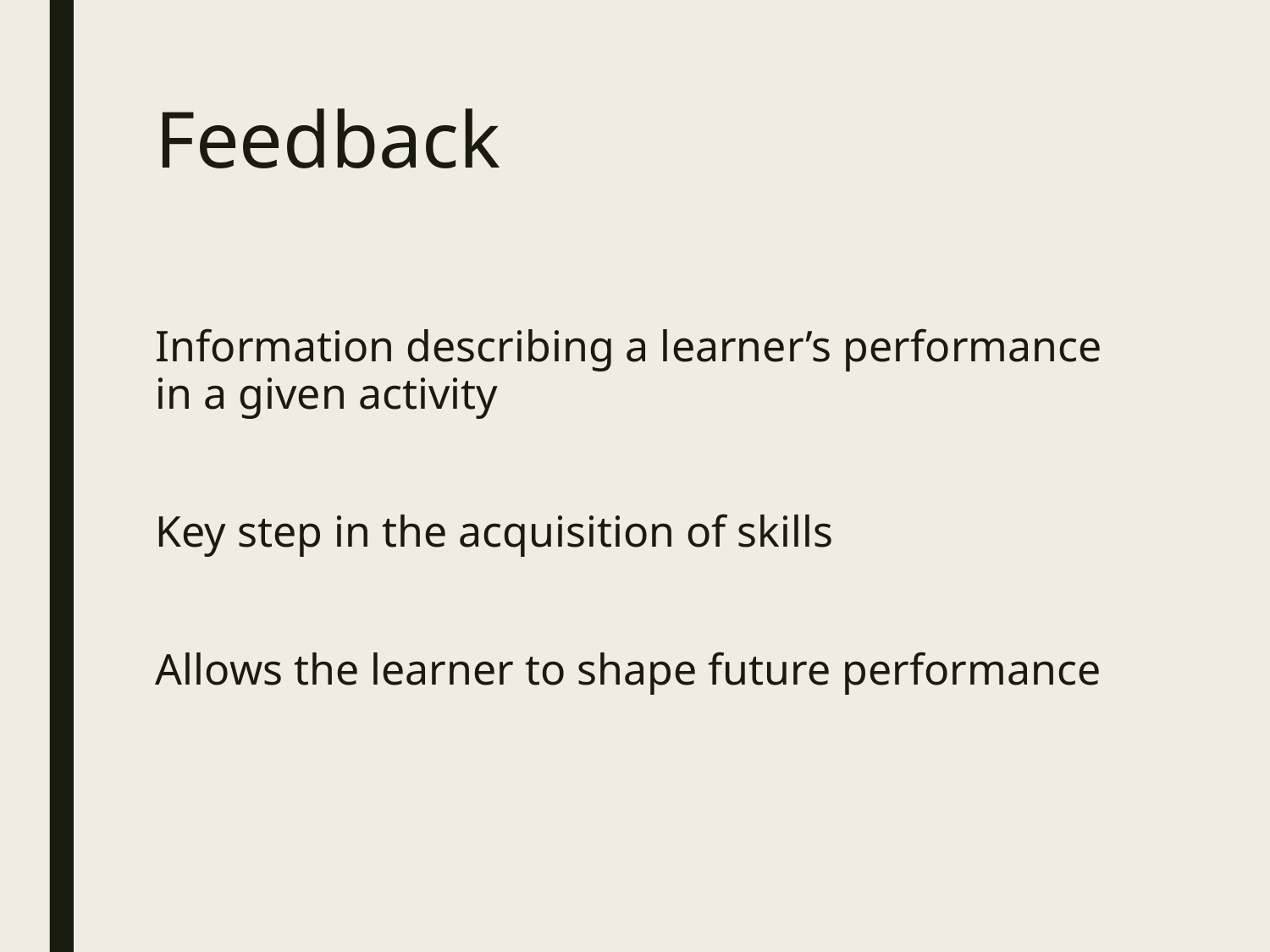

# Feedback
Information describing a learner’s performance in a given activity
Key step in the acquisition of skills
Allows the learner to shape future performance

## Slide 11
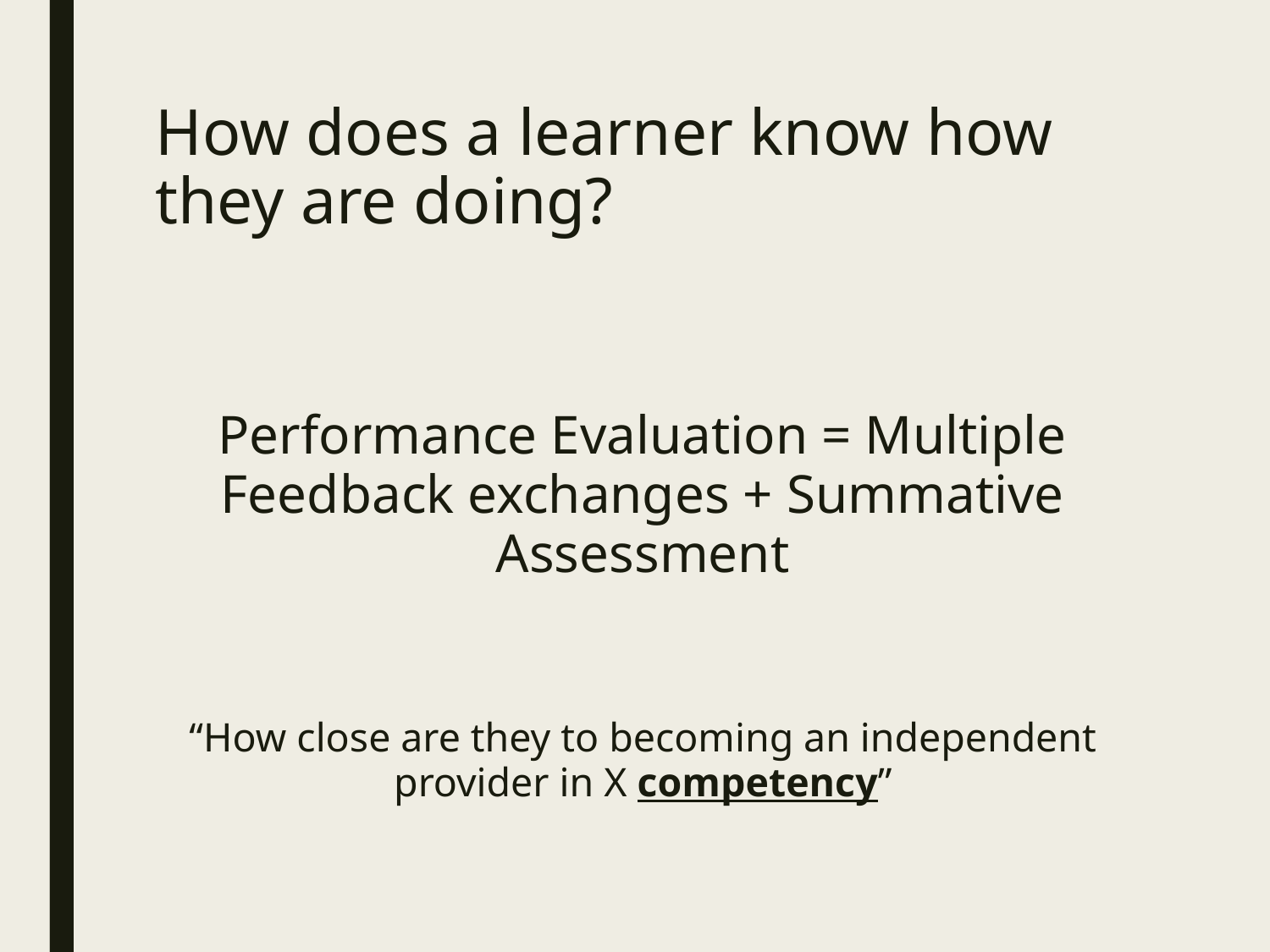

# How does a learner know how they are doing?
Performance Evaluation = Multiple Feedback exchanges + Summative Assessment
“How close are they to becoming an independent provider in X competency”

## Slide 12
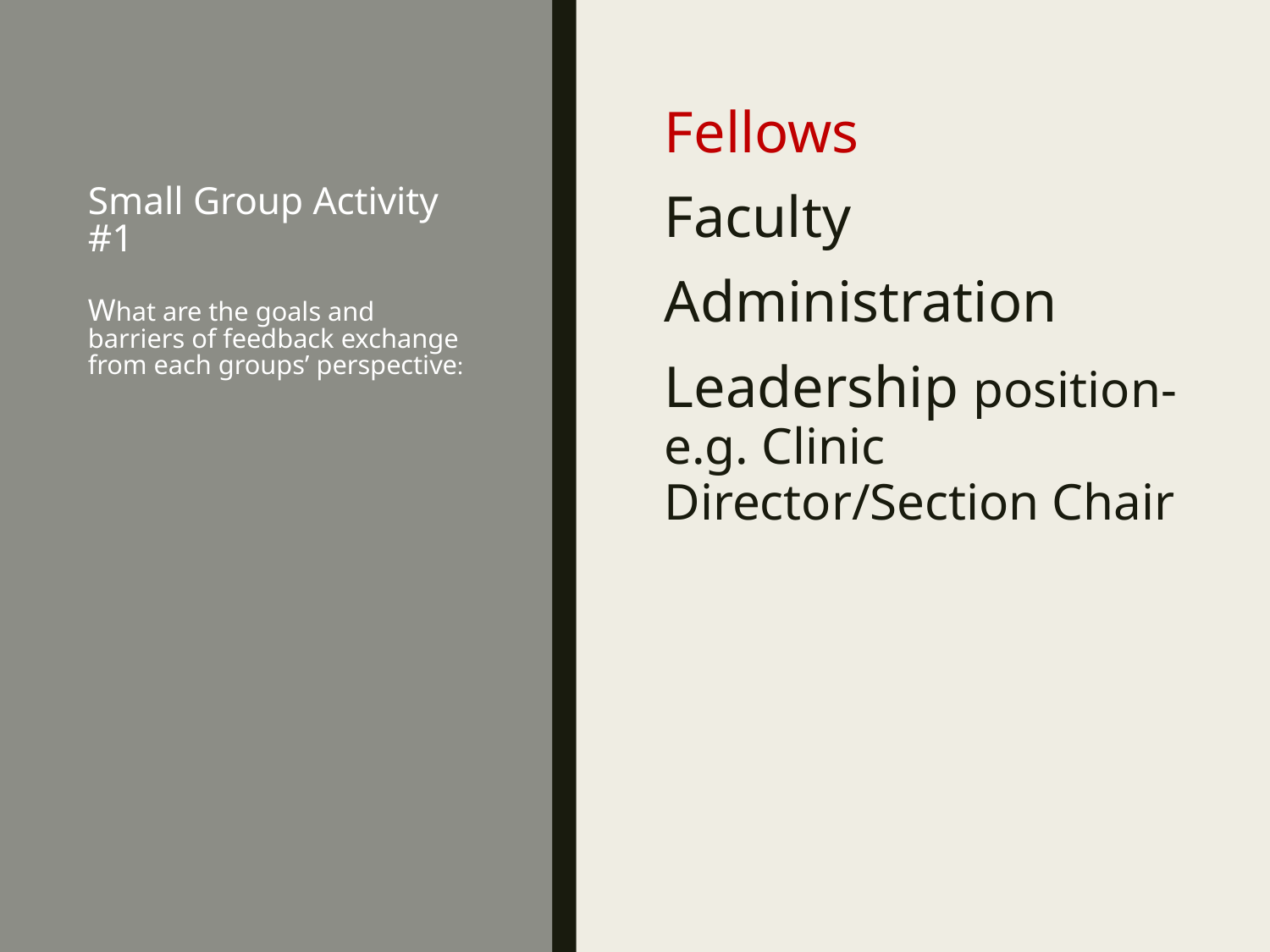

# Small Group Activity #1 What are the goals and barriers of feedback exchange from each groups’ perspective:
Fellows
Faculty
Administration
Leadership position-e.g. Clinic Director/Section Chair

## Slide 13
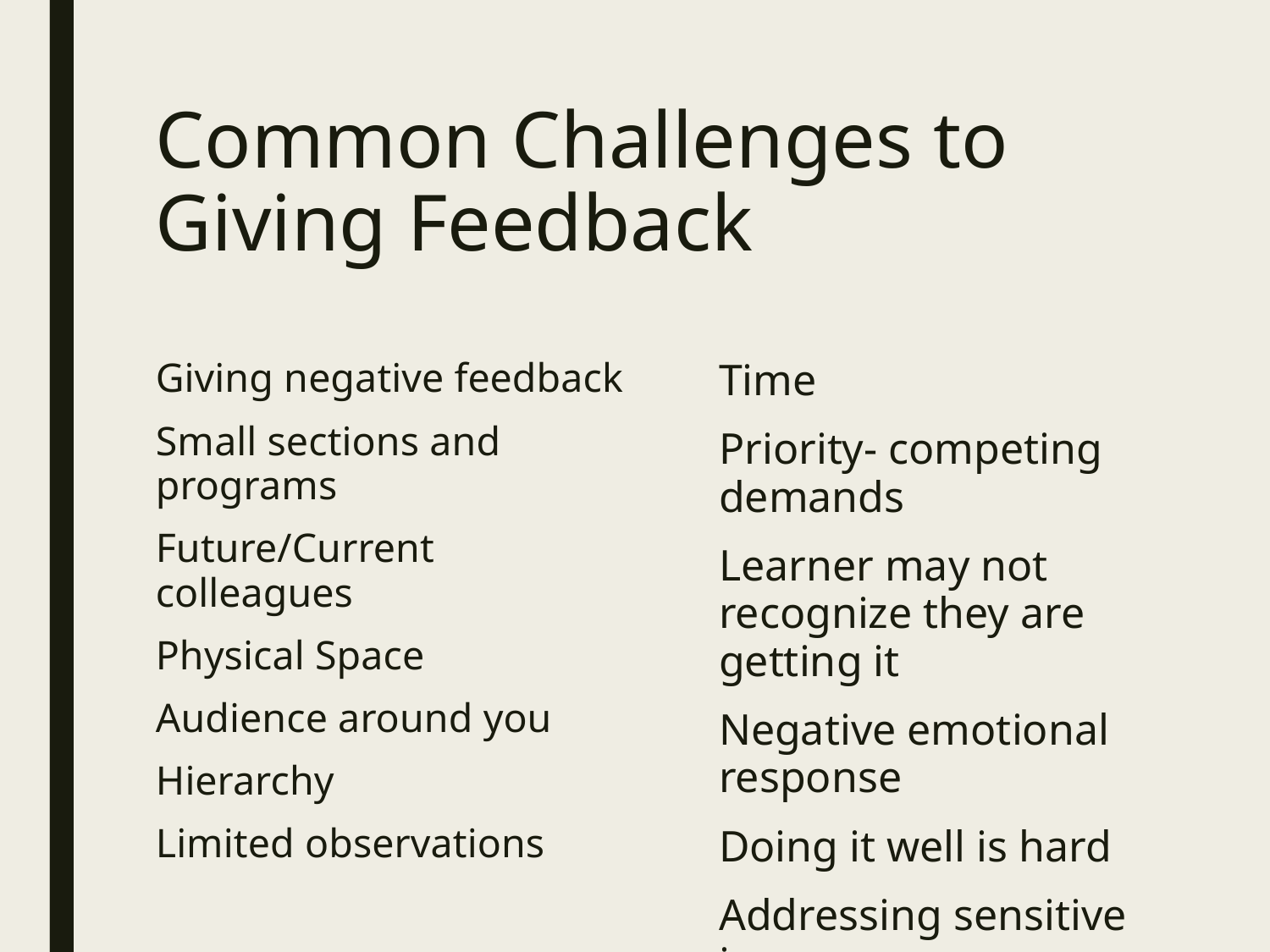

# Common Challenges to Giving Feedback
Time
Priority- competing demands
Learner may not recognize they are getting it
Negative emotional response
Doing it well is hard
Addressing sensitive issues
Giving negative feedback
Small sections and programs
Future/Current colleagues
Physical Space
Audience around you
Hierarchy
Limited observations

## Slide 14
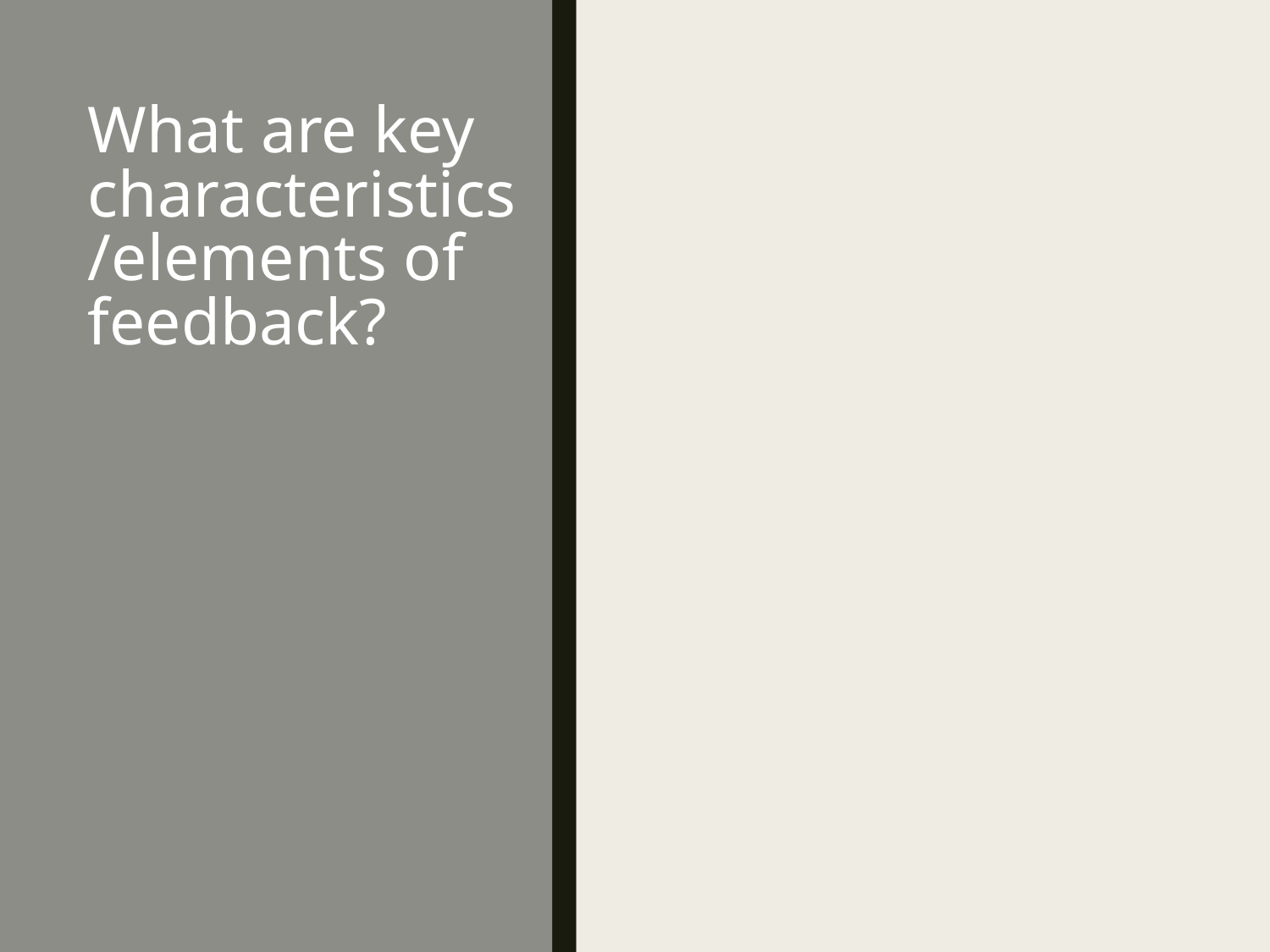

# What are key characteristics/elements of feedback?

## Slide 15
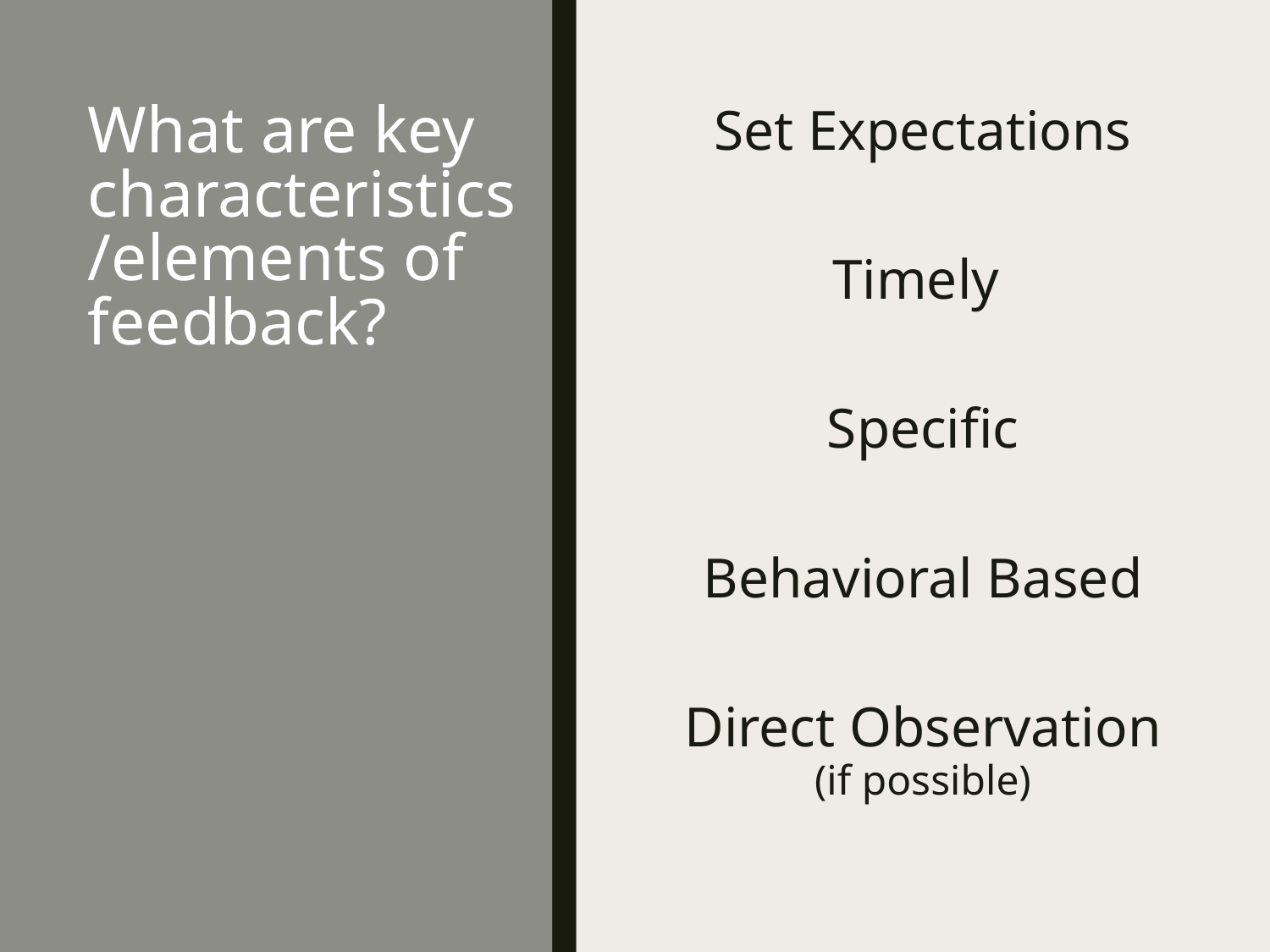

# What are key characteristics/elements of feedback?
Set Expectations
Timely
Specific
Behavioral Based
Direct Observation (if possible)

## Slide 16
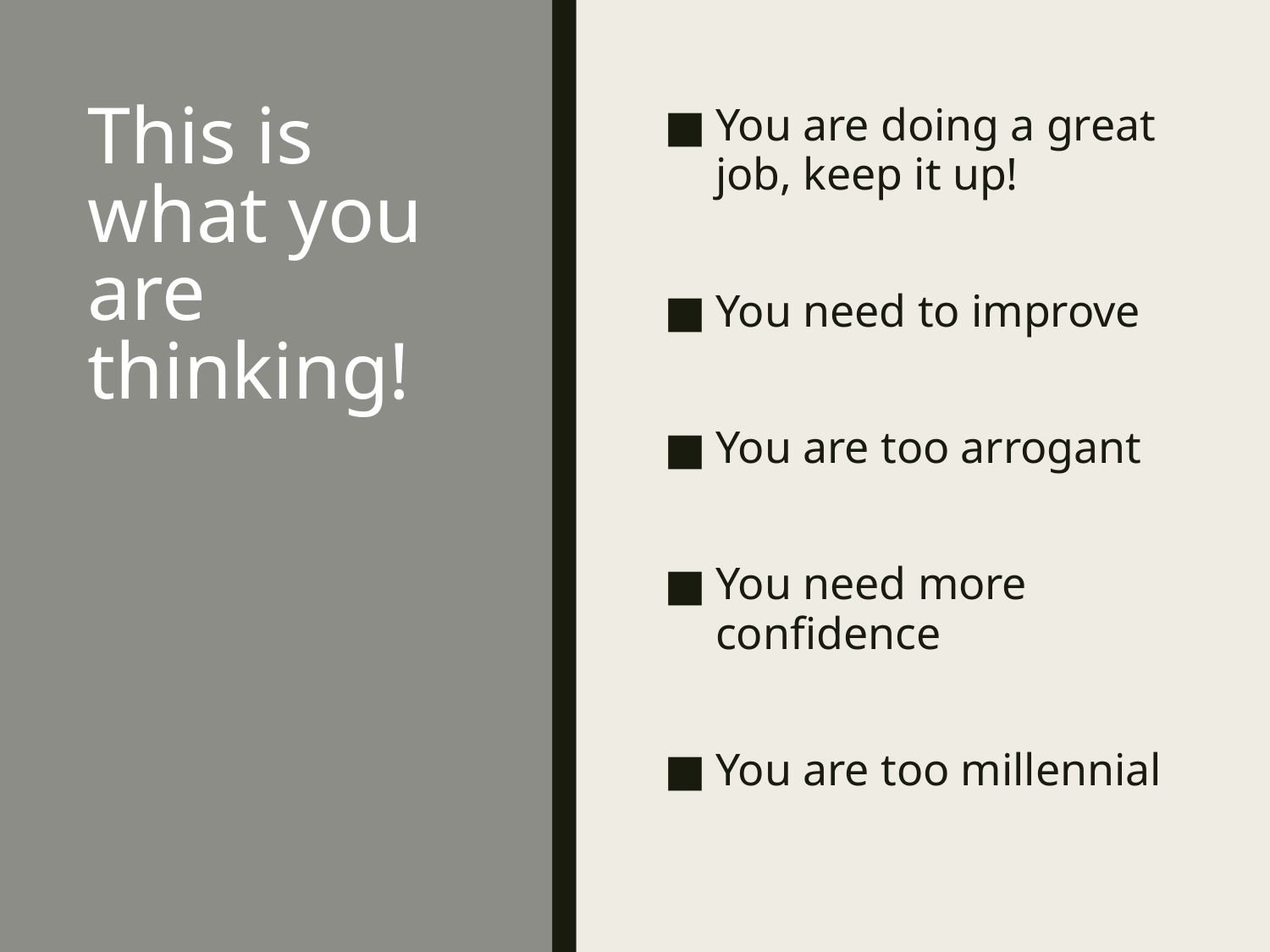

# This is what you are thinking!
You are doing a great job, keep it up!
You need to improve
You are too arrogant
You need more confidence
You are too millennial

## Slide 17
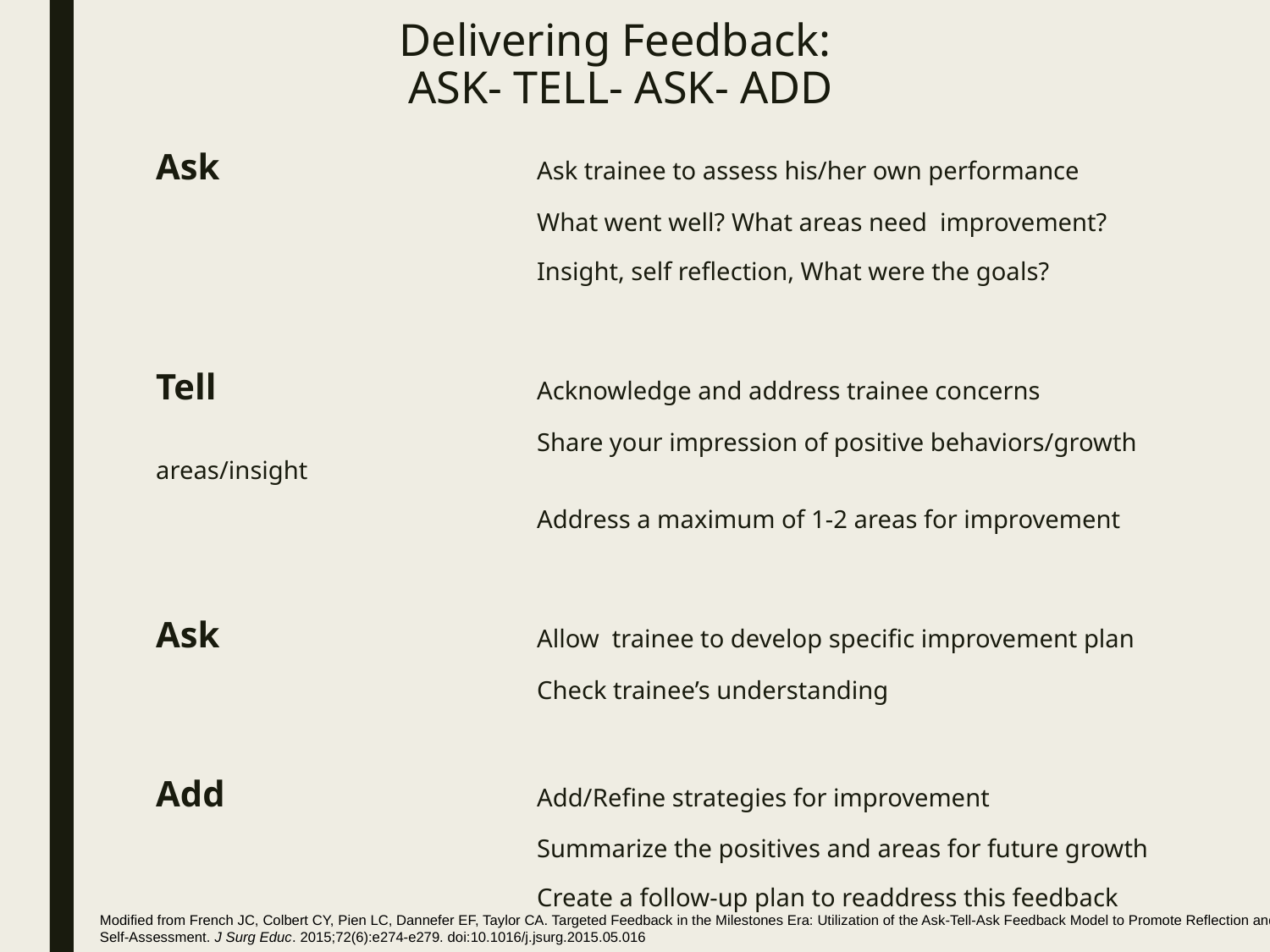

# Delivering Feedback: ASK- TELL- ASK- ADD
Ask 		Ask trainee to assess his/her own performance
			What went well? What areas need improvement?
			Insight, self reflection, What were the goals?
Tell			Acknowledge and address trainee concerns
			Share your impression of positive behaviors/growth areas/insight
 		 	Address a maximum of 1-2 areas for improvement
Ask		 	Allow trainee to develop specific improvement plan
			Check trainee’s understanding
Add			Add/Refine strategies for improvement
	 		Summarize the positives and areas for future growth
			Create a follow-up plan to readdress this feedback
Modified from French JC, Colbert CY, Pien LC, Dannefer EF, Taylor CA. Targeted Feedback in the Milestones Era: Utilization of the Ask-Tell-Ask Feedback Model to Promote Reflection and Self-Assessment. J Surg Educ. 2015;72(6):e274-e279. doi:10.1016/j.jsurg.2015.05.016

## Slide 18
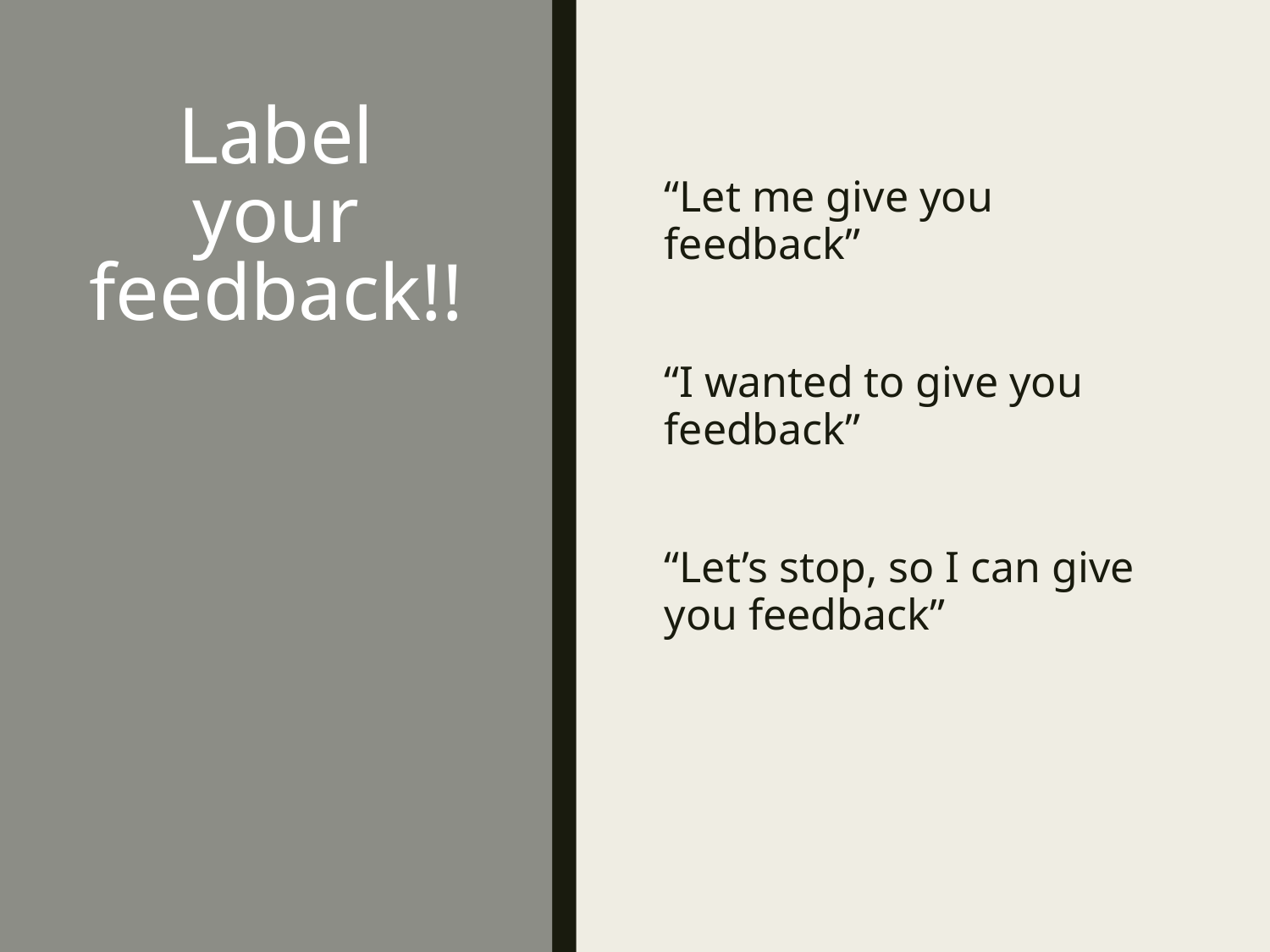

# Label your feedback!!
“Let me give you feedback”
“I wanted to give you feedback”
“Let’s stop, so I can give you feedback”

## Slide 19
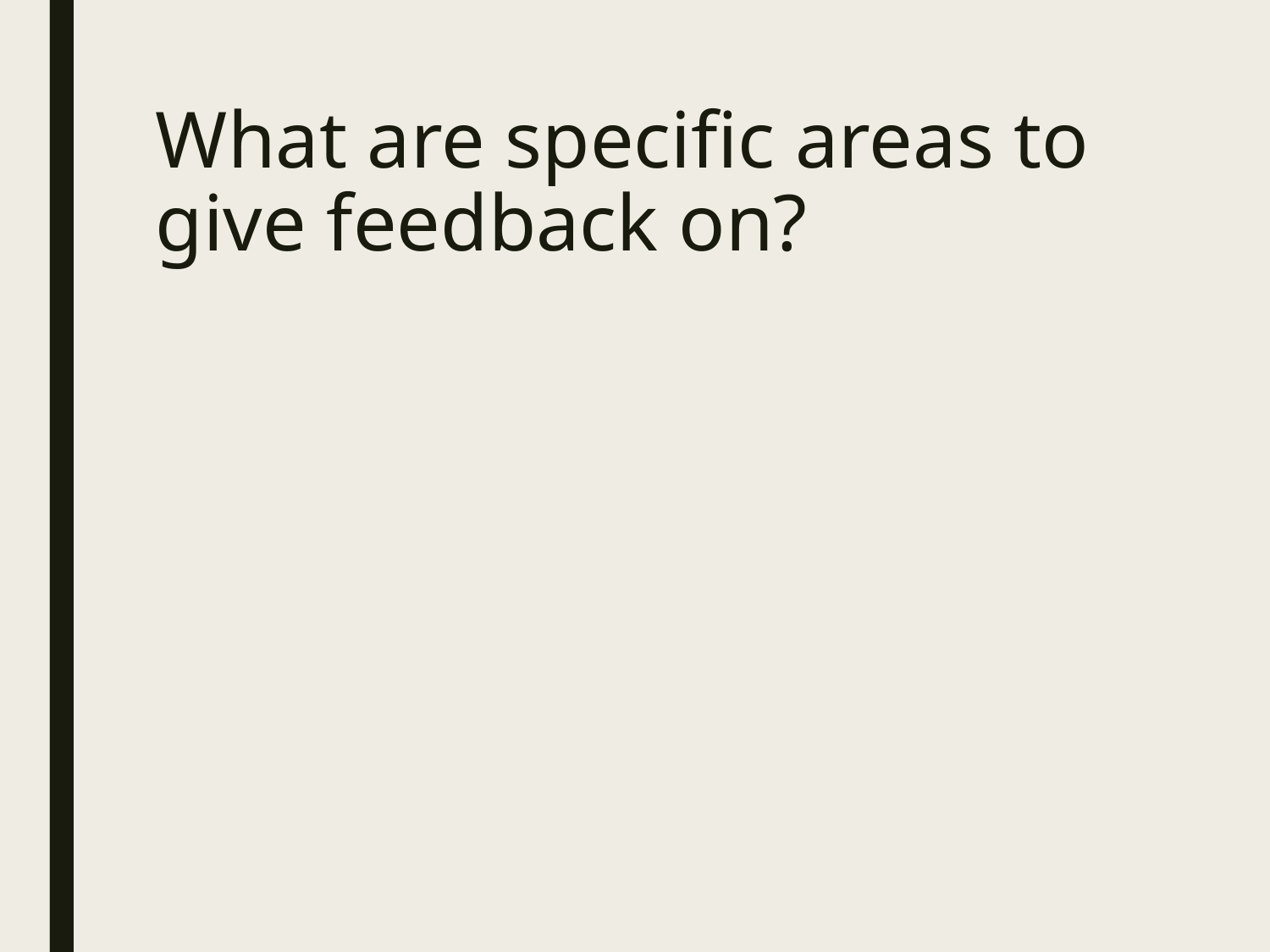

# What are specific areas to give feedback on?

## Slide 20
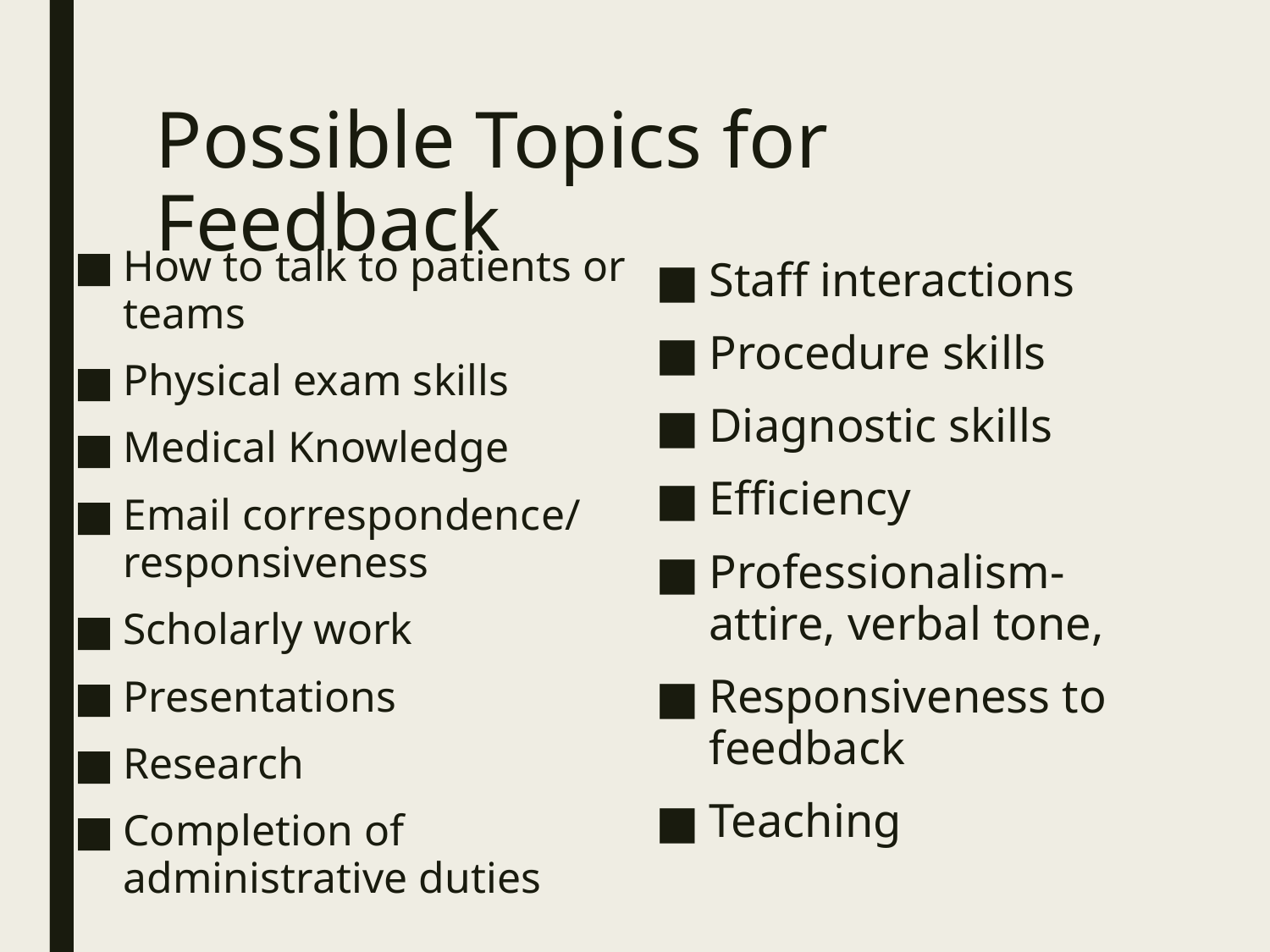

# Possible Topics for Feedback
How to talk to patients or teams
Physical exam skills
Medical Knowledge
Email correspondence/ responsiveness
Scholarly work
Presentations
Research
Completion of administrative duties
Staff interactions
Procedure skills
Diagnostic skills
Efficiency
Professionalism- attire, verbal tone,
Responsiveness to feedback
Teaching

## Slide 21
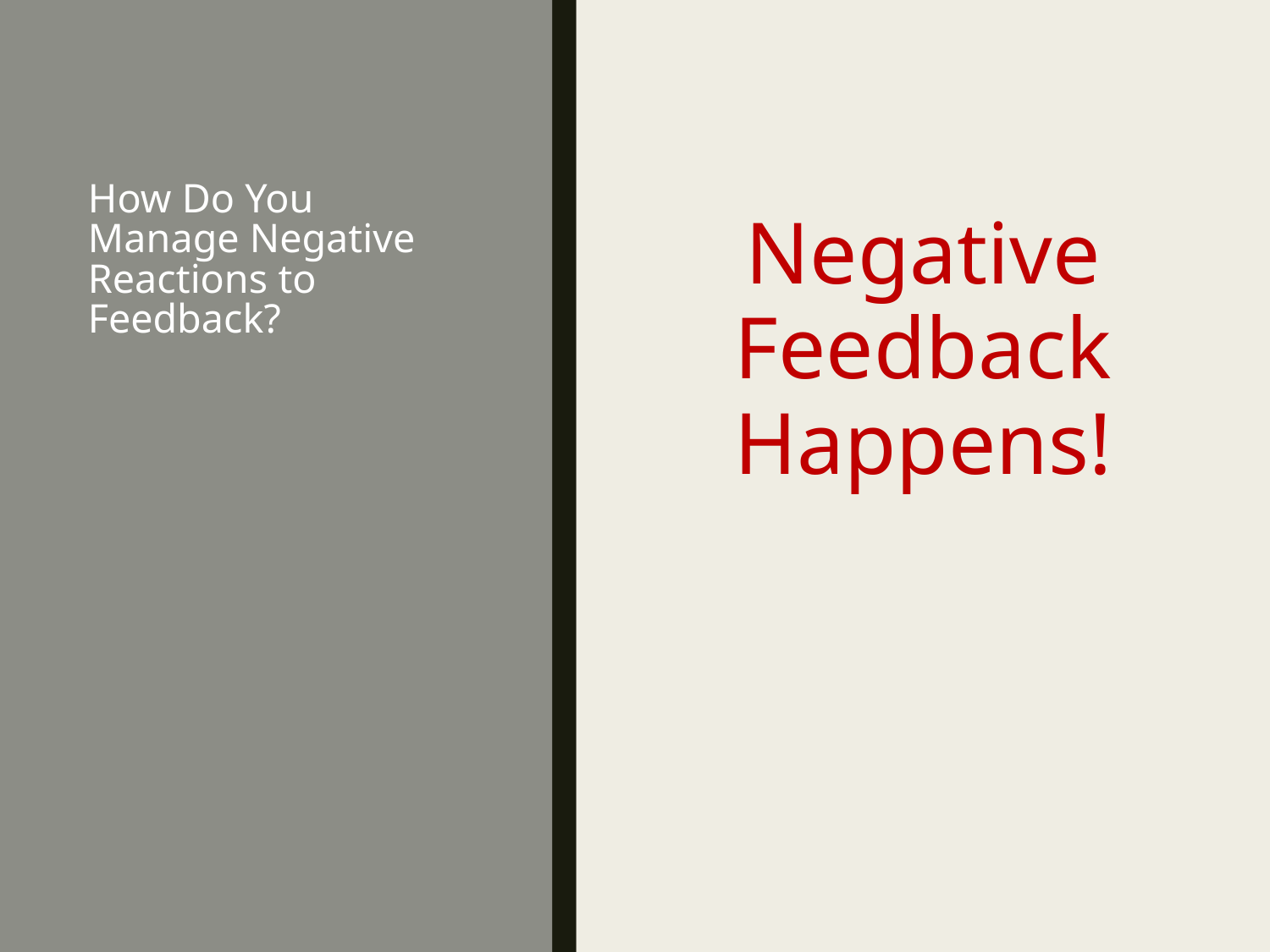

# How Do You Manage Negative Reactions to Feedback?
Negative Feedback Happens!

## Slide 22
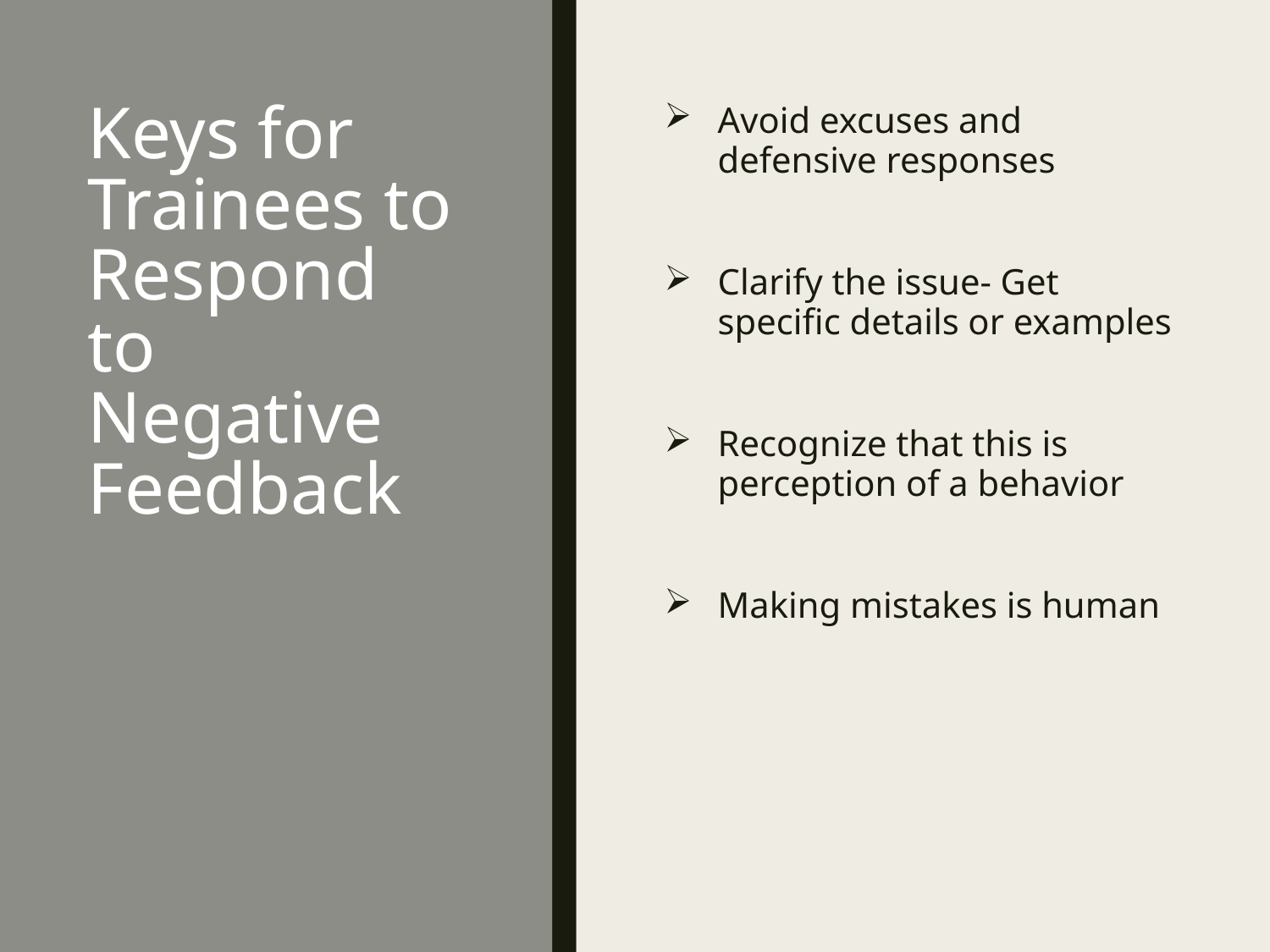

# Keys for Trainees to Respond to Negative Feedback
Avoid excuses and defensive responses
Clarify the issue- Get specific details or examples
Recognize that this is perception of a behavior
Making mistakes is human

## Slide 23
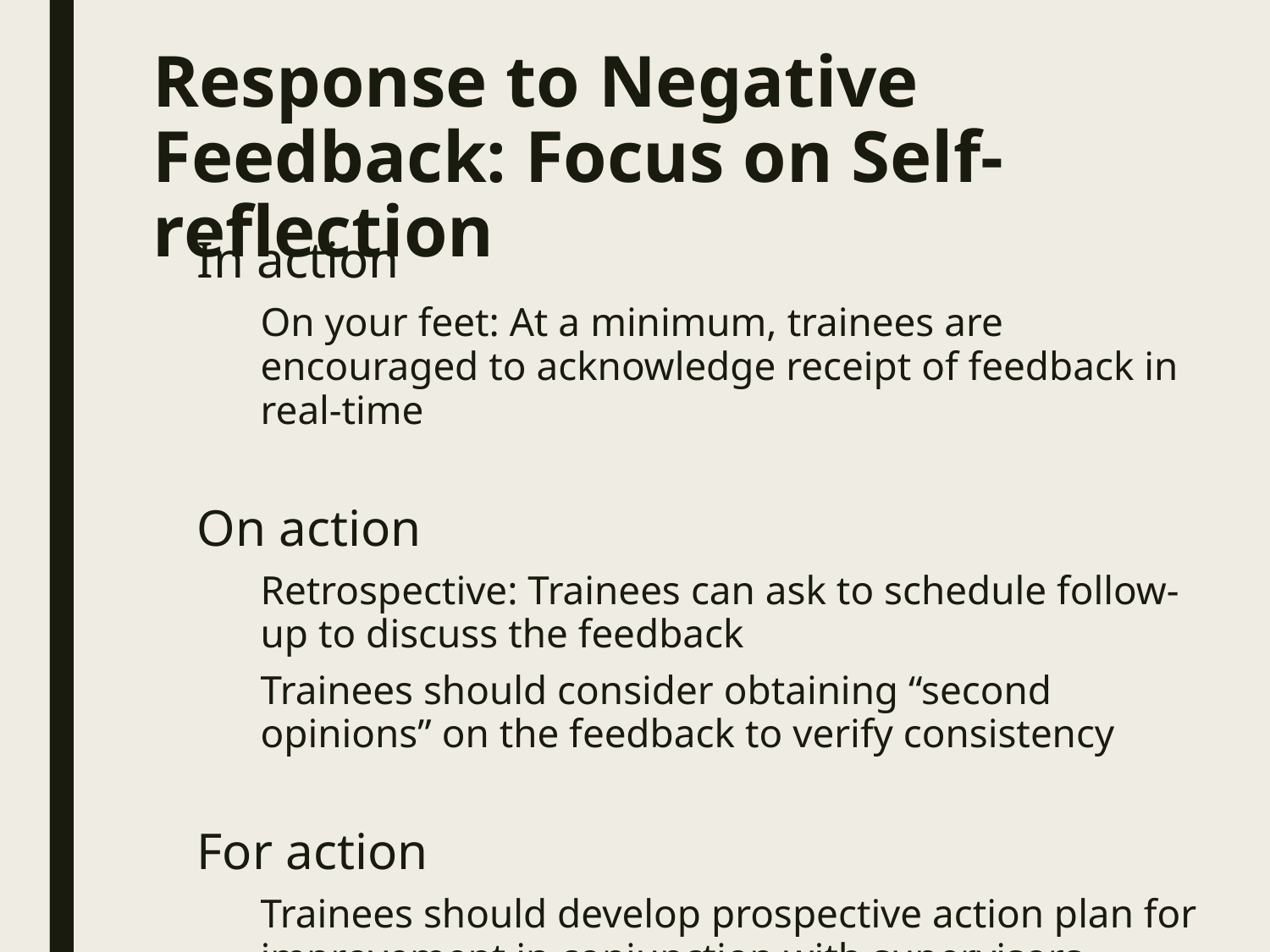

# Response to Negative Feedback: Focus on Self-reflection
In action
On your feet: At a minimum, trainees are encouraged to acknowledge receipt of feedback in real-time
On action
Retrospective: Trainees can ask to schedule follow-up to discuss the feedback
Trainees should consider obtaining “second opinions” on the feedback to verify consistency
For action
Trainees should develop prospective action plan for improvement in conjunction with supervisors

## Slide 24
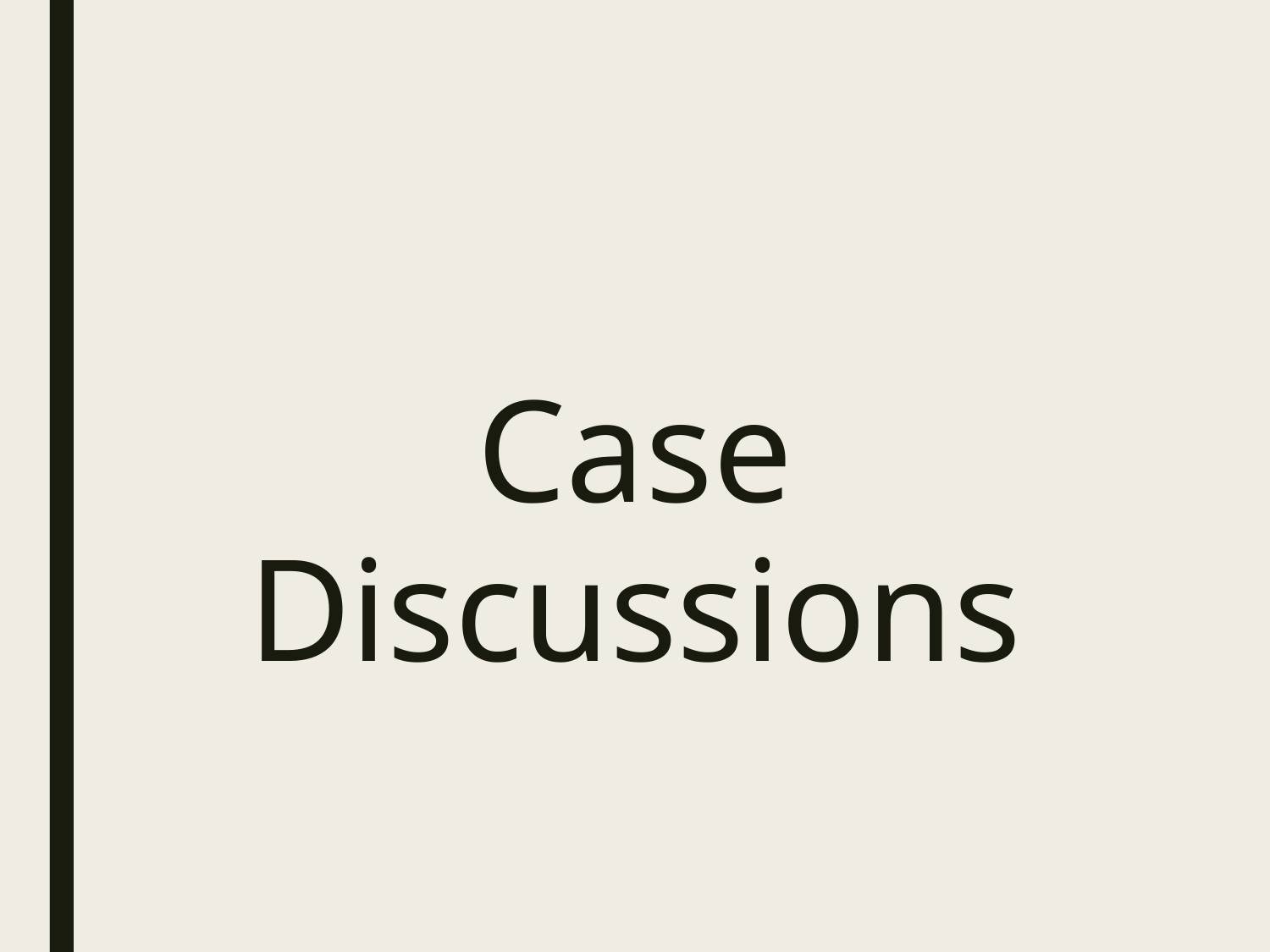

Case Discussions

## Slide 25
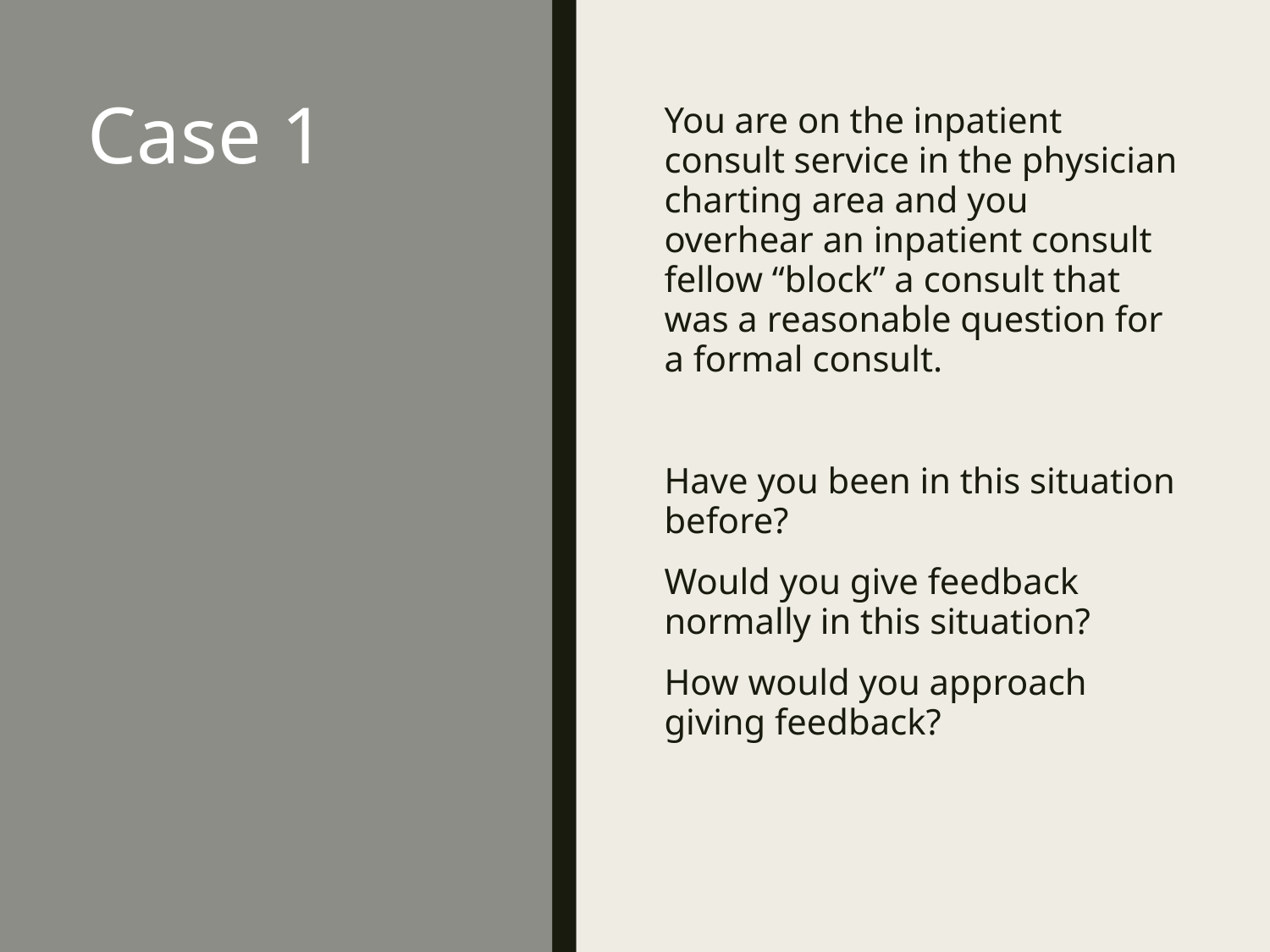

# Case 1
You are on the inpatient consult service in the physician charting area and you overhear an inpatient consult fellow “block” a consult that was a reasonable question for a formal consult.
Have you been in this situation before?
Would you give feedback normally in this situation?
How would you approach giving feedback?

## Slide 26
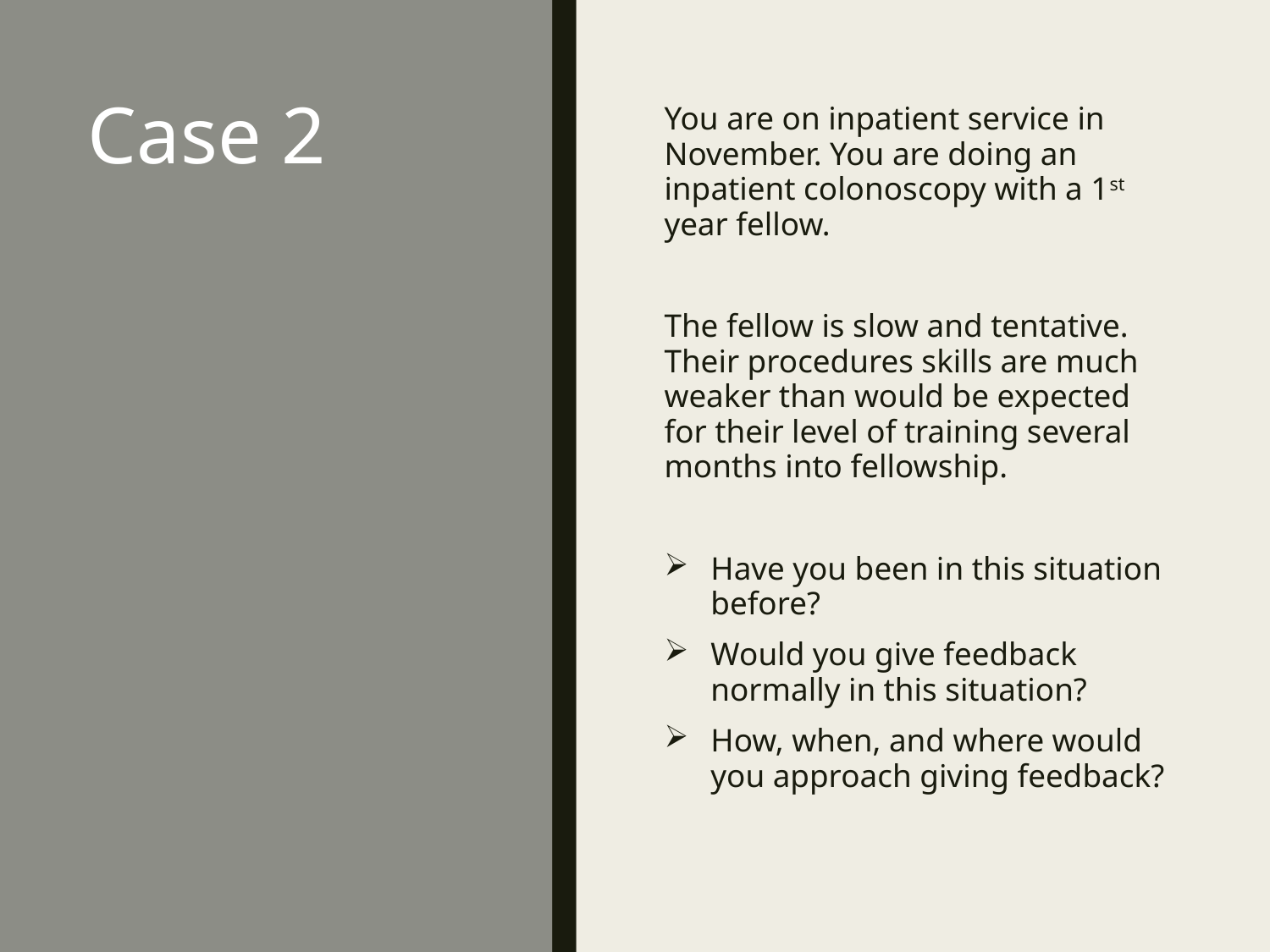

# Case 2
You are on inpatient service in November. You are doing an inpatient colonoscopy with a 1st year fellow.
The fellow is slow and tentative. Their procedures skills are much weaker than would be expected for their level of training several months into fellowship.
Have you been in this situation before?
Would you give feedback normally in this situation?
How, when, and where would you approach giving feedback?

## Slide 27
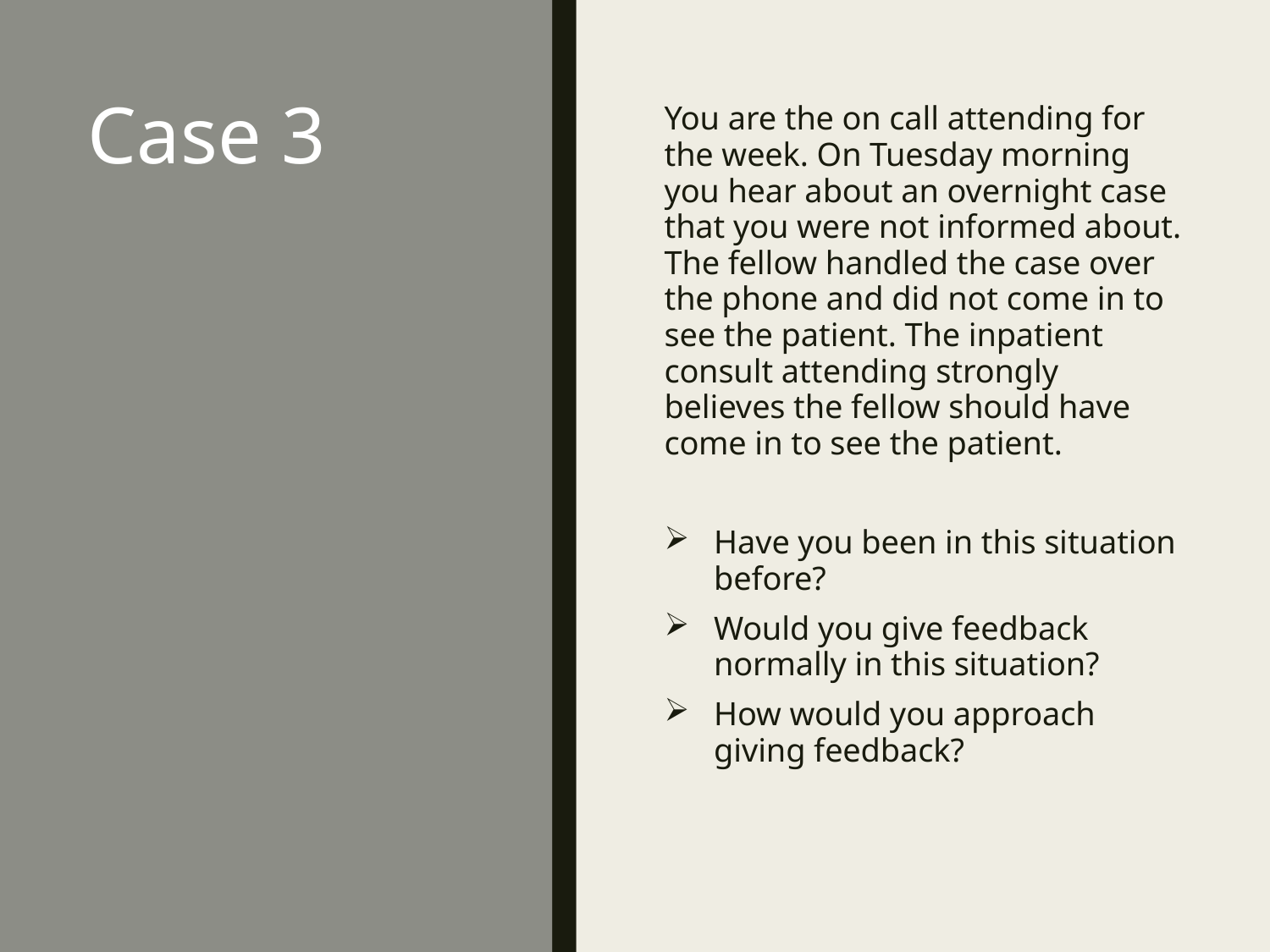

# Case 3
You are the on call attending for the week. On Tuesday morning you hear about an overnight case that you were not informed about. The fellow handled the case over the phone and did not come in to see the patient. The inpatient consult attending strongly believes the fellow should have come in to see the patient.
Have you been in this situation before?
Would you give feedback normally in this situation?
How would you approach giving feedback?

## Slide 28
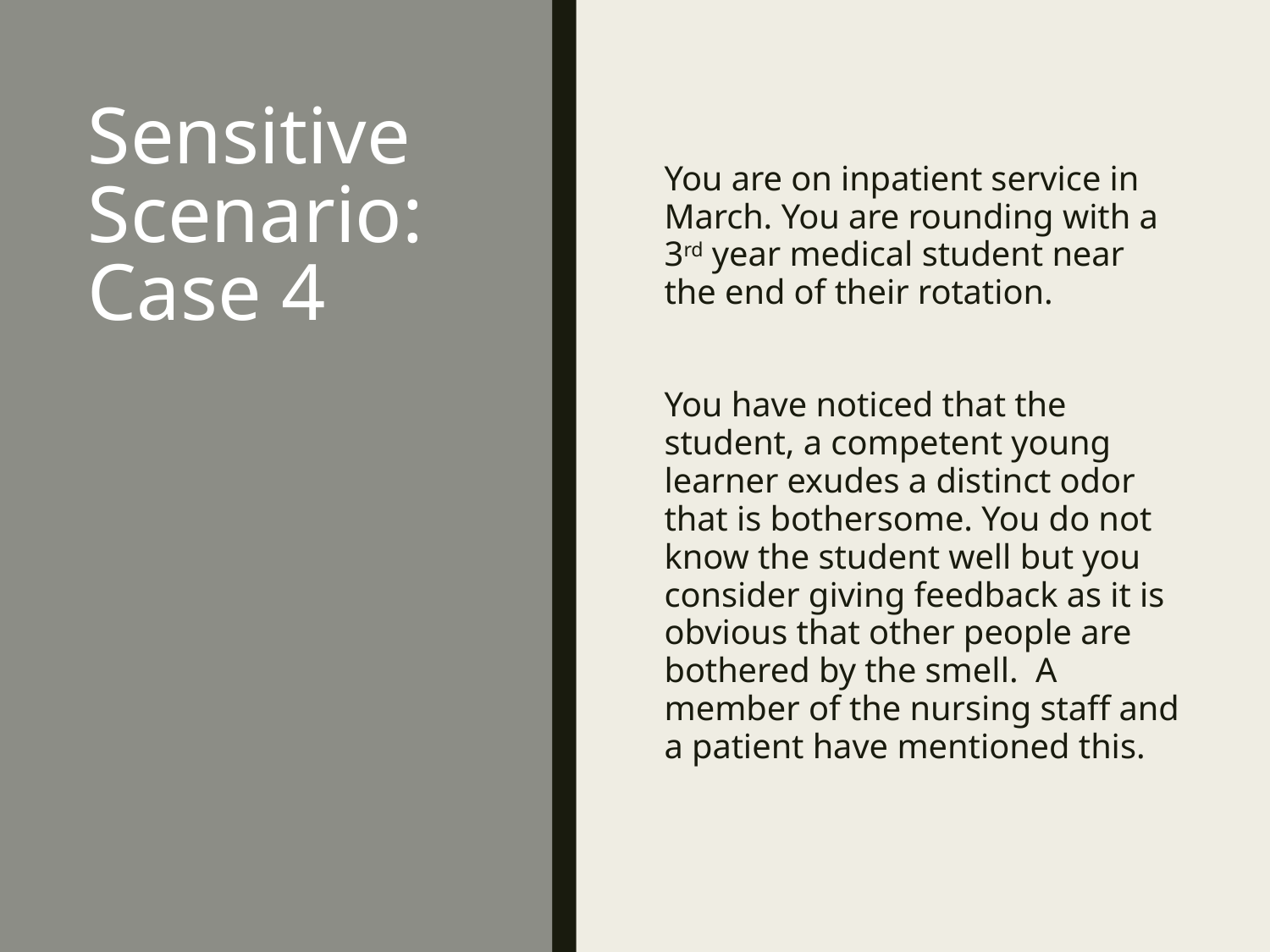

# Sensitive Scenario: Case 4
You are on inpatient service in March. You are rounding with a 3rd year medical student near the end of their rotation.
You have noticed that the student, a competent young learner exudes a distinct odor that is bothersome. You do not know the student well but you consider giving feedback as it is obvious that other people are bothered by the smell. A member of the nursing staff and a patient have mentioned this.

## Slide 29
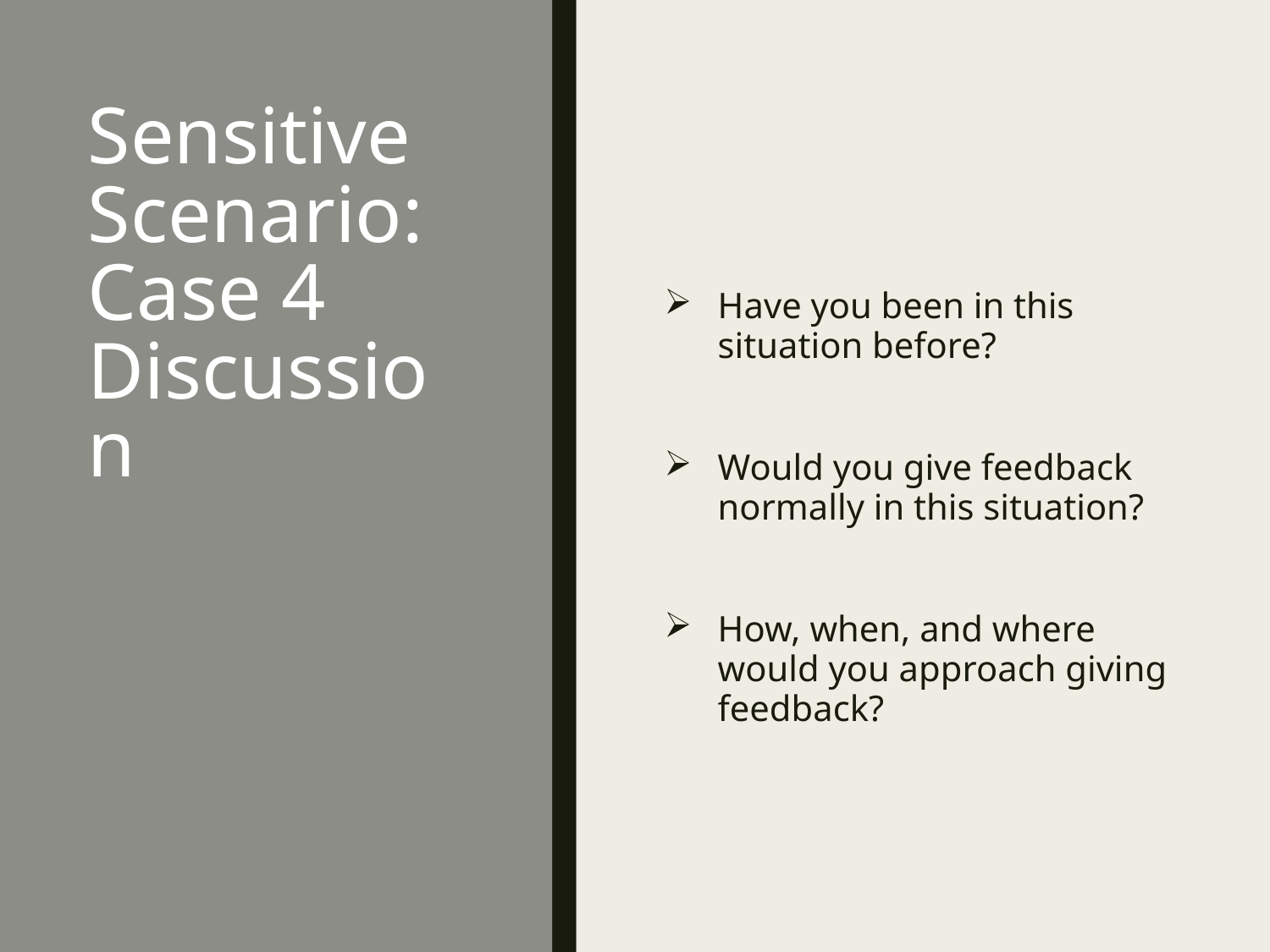

# Sensitive Scenario: Case 4 Discussion
Have you been in this situation before?
Would you give feedback normally in this situation?
How, when, and where would you approach giving feedback?

## Slide 30
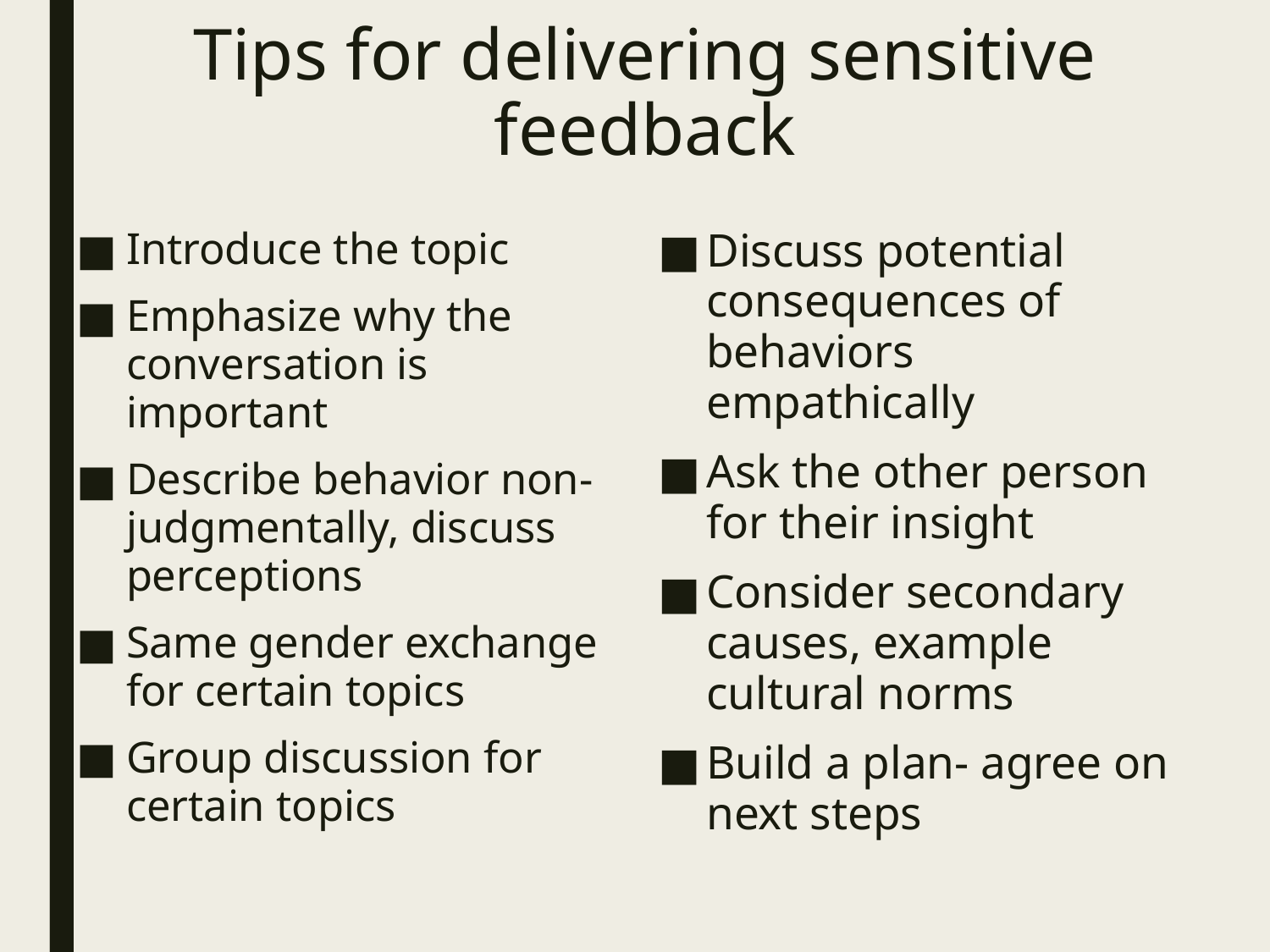

# Tips for delivering sensitive feedback
Introduce the topic
Emphasize why the conversation is important
Describe behavior non-judgmentally, discuss perceptions
Same gender exchange for certain topics
Group discussion for certain topics
Discuss potential consequences of behaviors empathically
Ask the other person for their insight
Consider secondary causes, example cultural norms
Build a plan- agree on next steps

## Slide 31
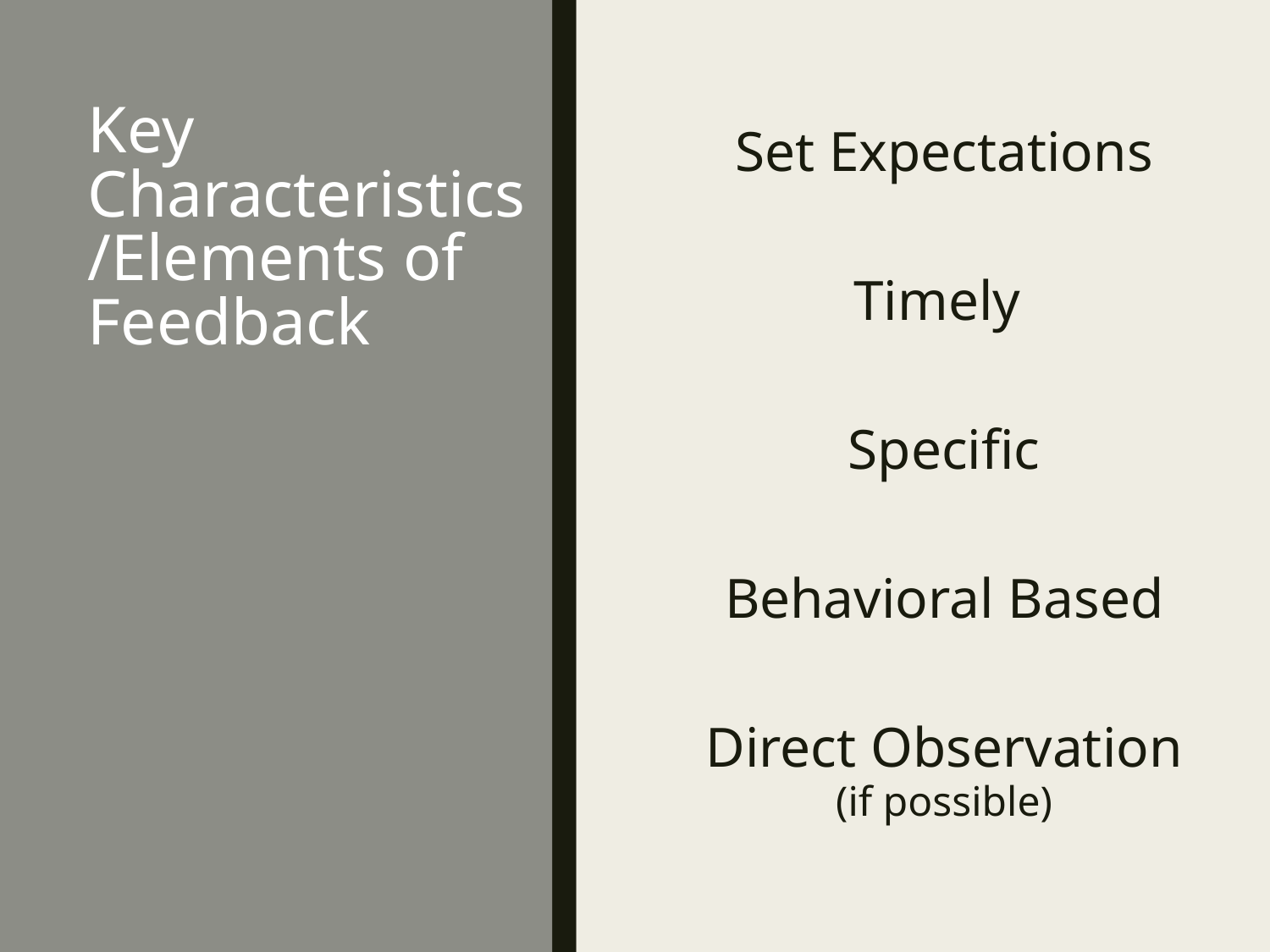

# Key Characteristics/Elements of Feedback
Set Expectations
Timely
Specific
Behavioral Based
Direct Observation (if possible)

## Slide 32
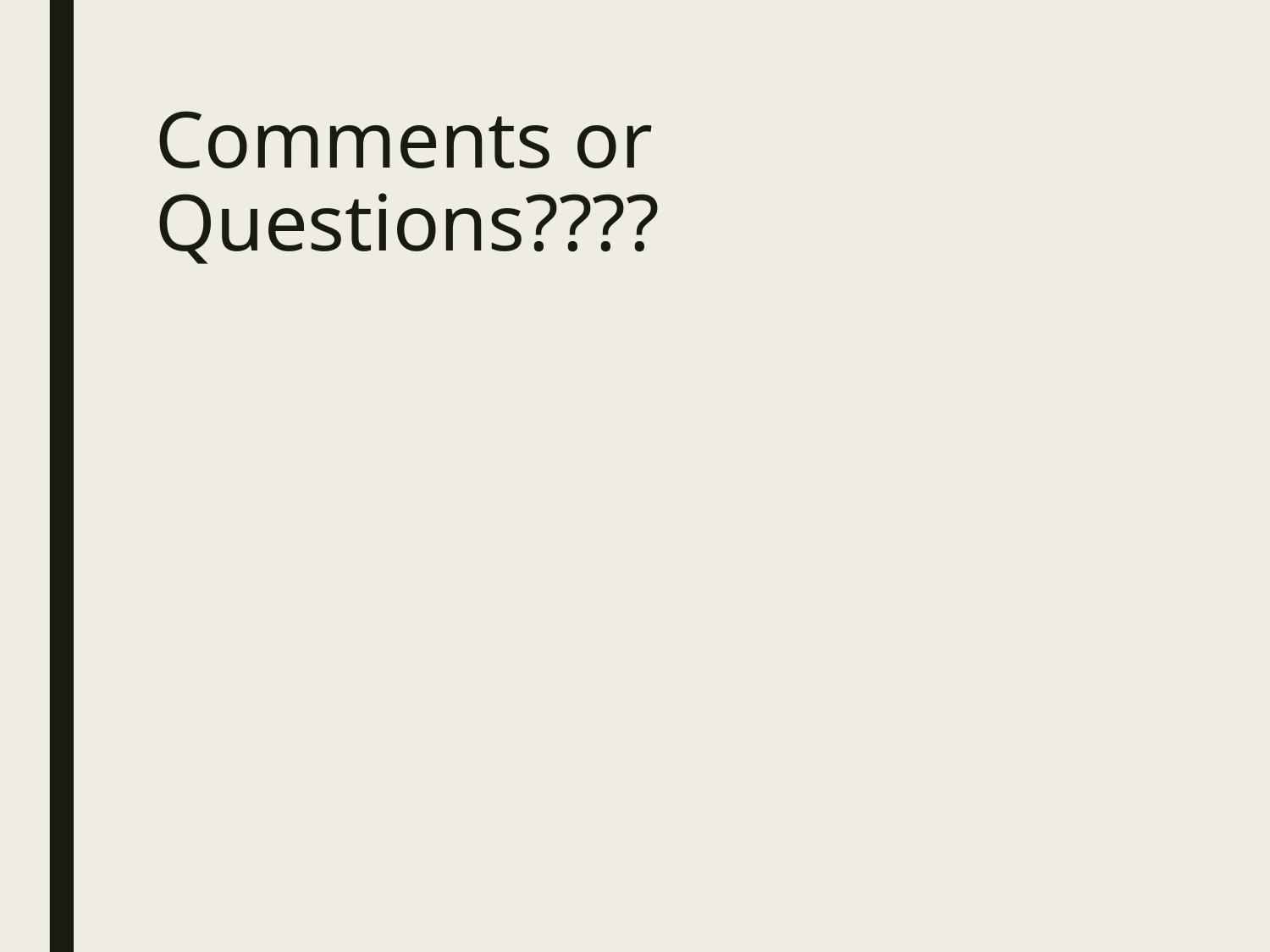

# Comments or Questions????
