# Supplementary material for: Can You Hear Me Now? Helping Faculty Improve Feedback Exchange for Internal Medicine Subspecialty Fellows
Source: MedEdPORTAL. 2021 Feb 17;17:11099. doi: 10.15766/mep_2374-8265.11099 (PMC7901254; doi:10.15766/mep_2374-8265.11099)
Supplement: Supplementary file 1 — Facilitators Guide.docxPowerPoint.pptxPreworkshop Survey.docPostworkshop Survey.doc [file mep_2374-8265.11099-s001.zip › C. Preworkshop Survey.doc]

|  | |  | |
| --- | --- | --- | --- |
| **Feedback Faculty Pre-Workshop Assessment** | | | |
| **Activity Title:** | **Feedback** | **Surveyors:** |  |

**Unique Identifier:**

**First 2 letters of mother’s maiden name + month of birth as 2 digit number + first letter of the city you were born in**

**(Eg: Mothers maiden name is Smith, Born in December, and birth city Boston: unique identifier is “Sm12b”)**

**Enter your unique identifier here: _________________________**

1. What gender do you identify with (circle)	M	F	Other (specify) ____________
2. # years since finishing training	______________________
3. Professional Track (circle)	Clinician	Basic scientist	Clin scientist	Clin educator
4. **How comfortable are you with giving feedback to trainees (circle):**

| **1** | **2** | **3** | **4** | **5** |
| --- | --- | --- | --- | --- |
| Extremely uncomfortable | Somewhat uncomfortable | Average | Somewhat comfortable | Extremely comfortable |

**What barriers, besides time, do you encounter in regards to receiving and giving feedback with trainees?**
