# Supplementary material for: Can You Hear Me Now? Helping Faculty Improve Feedback Exchange for Internal Medicine Subspecialty Fellows
Source: MedEdPORTAL. 2021 Feb 17;17:11099. doi: 10.15766/mep_2374-8265.11099 (PMC7901254; doi:10.15766/mep_2374-8265.11099)
Supplement: Supplementary file 1 — Facilitators Guide.docxPowerPoint.pptxPreworkshop Survey.docPostworkshop Survey.doc [file mep_2374-8265.11099-s001.zip › D. Postworkshop Survey.doc]

|  | |  | |
| --- | --- | --- | --- |
| **Feedback Faculty Post-Workshop Assessment** | | | |
| **Activity Title:** | **Feedback** | **Surveyors:** |  |

**Unique Identifier:**

**First 2 letters of mother’s maiden name + month of birth as 2 digit number + first letter of the city you were born in**

**(Eg: Mothers maiden name is Smith, Born in December, and birth city Boston: unique identifier is “Sm12b”)**

**Enter your unique identifier here: _________________________**

1. ***AFTER*** this session, how comfortable are you with giving feedback to trainees (circle):

| **1** | **2** | **3** | **4** | **5** |
| --- | --- | --- | --- | --- |
| Extremely uncomfortable | Somewhat uncomfortable | Average | Somewhat comfortable | Extremely comfortable |

**Please list 2 things you are taking away from this session:**

**A.**

**B.**

**Comments/Suggestions:**
